# Feasibility, safety and tolerability of intradialytic creatine supplementation in hemodialysis: A double-blind, randomized, placebo-controlled pilot study

**DOI:** 10.1371/journal.pone.0354883

**Published:** 2026-08-05

**Authors:** Caecilia S. E. Doorenbos, Yvonne van der Veen, Adrian Post, Jip Jonker, Tim J. Knobbe, Daan Kremer, Dion Groothof, M. Rebecca Heiner-Fokkema, Christa A. Koops, Klaas Bijsterveld, Erik Marsman, Ietje T. Hazenberg, Nienke A. Manson, Theo Y. J. Appeldoorn, Hendrikus H. Boersma, Bart G. J. Dekkers, Daan J. Touw, Theo Wallimann, Casper F. M. Franssen, Stephan J. L. Bakker

**Affiliations:** 1 University of Groningen, Department of Internal Medicine, Division of Nephrology, University Medical Center Groningen, Groningen, The Netherlands; 2 University of Groningen, Department of Laboratory Medicine, University Medical Center Groningen, Groningen, The Netherlands; 3 Dialysis Center Groningen, Groningen, The Netherlands; 4 University of Groningen, Department of Clinical Pharmacy and Pharmacology, University Medical Center Groningen, Groningen, The Netherlands; 5 Department of Biology, ETH Zurich, Zurich, Switzerland; Dynamical Business & Science Society - DBSS International SAS, COLOMBIA

## Abstract

**Background:**

Patients receiving hemodialysis are at risk of creatine deficiency due to impaired endogenous synthesis and losses during dialysis. Creatine deficiency is associated with lower health-related quality of life and higher mortality. We investigated the feasibility, tolerability and safety of intradialytic creatine supplementation during hemodialysis.

**Methods:**

In this double-blind, randomized, placebo-controlled pilot study (ClinicalTrials.gov ID: NCT05148208), 16 hemodialysis patients were included into four sequentially initiated groups, with each group receiving a fixed creatine concentration in the dialysate (0.5 mmol/L, 1.0 mmol/L, 1.5 mmol/L and 2.0 mmol/L, respectively), with block randomization in a 3:1 ratio (creatine:placebo) per group. Dropouts were replaced. Intra-dialytic creatine was administered during a 6-week period. Primary outcomes were changes in pre-dialytic intra-erythrocytic and plasma creatine concentrations, which were analyzed with linear mixed models.

**Results:**

16 hemodialysis patients (aged 27–78 years, 9 female) completed the study. Creatine supplementation was generally well tolerated. One patient receiving 1.5 mmol/L creatine dropped out due to a potential side effect. No serious adverse events occurred. The median (minimum-maximum) baseline pre-dialytic intra-erythrocytic and plasma creatine concentrations were 963 (407–1822) and 35 (13–287) µmol/L, respectively. At 6 weeks, the changes in pre-dialytic intra-erythrocytic creatine concentration compared to placebo (95% CI) were +399 (−269, 1066), + 685 (+17, + 1352), + 1146 (+431, + 1766) and +1098 (+431, + 1766) µmol/L for the increasing dosage treatment groups respectively (overall treatment effect p < 0.001). The changes in pre-dialytic plasma creatine concentration compared to placebo were −16 (−301, + 270), + 21 (−264, + 307), + 119 (−264, + 307) and +163 (−122, + 449) µmol/L for the increasing dosage treatment groups respectively (overall treatment effect p = 0.002).

**Conclusion:**

Intra-dialytic creatine supplementation is a feasible, safe, well-tolerated method to increase circulating creatine concentrations in patients treated with hemodialysis. These findings support the rationale for a larger randomized controlled trial to investigate its potential to improve health-related quality of life and reduce mortality risk in this population.

**Trial Registration:** ClinicalTrials.gov ID NCT05148208, date of first registration 08/12/2021

## 1. Introduction

Kidney failure is a leading cause of morbidity and mortality worldwide [1]. Although lifesaving, dialysis is associated with lower health-related quality of life (HRQoL) compared to the general population, as well as a lower life expectancy [2]. We hypothesize that creatine deficiency is a modifiable risk factor underlying low HRQoL and low life expectancy in patients treated with dialysis [3–5].

Creatine, a natural nitrogenous organic acid, plays a crucial role in energy metabolism and is vital for proper cell functioning. ~ 90% of the body content of creatine is found in muscle and nervous tissue [6,7]. Approximately 1.7% of the total creatine pool is lost daily through spontaneous, non-enzymatic conversion into its waste product creatinine, which is excreted in urine under healthy conditions and into the dialysate in patients treated with hemodialysis. Next to conversion to creatinine, circulating creatine is additionally lost directly into the dialysate, due to the permeability of the dialysis filter to both creatinine and creatine [3,5]. The continuous loss of creatine necessitates replenishment of the creatine pool, through endogenous creatine synthesis and dietary intake [6]. Endogenous creatine synthesis involves a rate-limiting step, primarily situated in the kidney, where the enzyme glycine amidino-transferase (GATM) converts arginine and glycine into the creatine precursor guanidinoacetate [7–9]. Subsequently, guanidinoacetate is converted to creatine by methylation in the liver [6]. With normal kidney function, the capacity of AGAT will usually enable sufficient endogenous creatine synthesis to replenish the daily losses. However, it is highly likely that individuals with kidney failure cannot produce sufficient creatine and depend on dietary creatine [3,4]. Decreased endogenous synthesis, losses into the dialysate and often low dietary creatine intake, increase risk for creatine deficiency in patients on hemodialysis [3–5,8–12]. This is supported by our observations that hemodialysis patients have low plasma creatine concentrations compared with the general population [13], which was associated with having low muscle mass, hypoalbuminemia, and severe fatigue [5]. Therefore, patients treated with hemodialysis may benefit from creatine supplementation [3].

Because of its performance-enhancing capabilities, oral creatine supplementation has been studied extensively in athletes [14], but only few studies have been conducted in patients treated with hemodialysis [15,16]. Importantly, oral creatine supplementation has drawbacks, including poor oral tolerance in some individuals, co-ingestion of high volumes of water and unopposed losses of creatine into the dialysate. Intradialytic application of creatine supplementation would not have these drawbacks [3–5]. Furthermore, no harms are expected when applying intradialytic creatine supplementation, although a minor increase in total body water may occur after creatine supplementation, which is thought to appear mainly intracellularly [17].

In this study, we aimed to investigate the safety, feasibility and tolerability of intradialytic creatine supplementation in patients treated with hemodialysis by continuously adding a creatine monohydrate concentrate to the dialysate. Furthermore, we aimed to find the most effective and tolerable dose for intradialytic creatine supplementation.

## 2. Materials and methods

### 2.1 Study design

The study design of this randomized, double-blind, placebo-controlled study in patients treated with hemodialysis has been registered (ClinicalTrials.gov ID: NCT05148208, date of first registration: 08/12/2021) and has previously been described in detail [13]. The study was approved by the local Medical Ethical Committee of the University Medical Center Groningen (UMCG) (2022, NL79248.042.21) and was conducted in the University Medical Center Groningen (UMCG) and in the Dialysis Center Groningen (DCG). The study was performed consistent with the Declaration of Helsinki. Signed informed consent was obtained from all participants. The study was conducted according to the CONSORT Harms 2022 guidelines.

### 2.2 Participants

Participants were recruited between January 5^th^, 2023 and January 11^th^, 2024 and the study was conducted between August 3^rd^, 2023 and March 21^st^, 2024. The delay between initial recruitment and study initiation was due to a technical problem in the production facility of the investigational product. Inclusion criteria were age ≥ 18 years, hemodialysis treatment in the UMCG or DCG, hemodialysis vintage ≥2 months and signed informed consent. Exclusion criteria were pregnancy, presence of infection, active malignancies (excluding non-metastasized non-melanoma dermatological malignancies), insufficient proficiency in the Dutch language, life expectancy <1 year (determined by treating nephrologist), kidney transplantation planned within the study duration, inability to complete questionnaires, and average hemoglobin level at three previous monthly routine assessments <5.5 mmol/L.

### 2.3 Randomization and blinding

Block randomization with a 3:1 creatine-to-placebo ratio was performed within four dose-escalation groups, each consisting of four participants. The sequential groups each received an incrementally higher creatine dose, starting with the lowest dose and proceeding to the highest dose.

Before initiation of patient inclusion, study identification numbers were assigned per group-specific randomization block (1–4, 5–8, 9–12, and 13–16). For each block, a pharmacist from the Department of Clinical Pharmacy and Pharmacology (UMCG), who was not involved in study execution, randomly assigned one number to placebo treatment and the remaining three to creatine treatment. The treatment allocations for the study identification numbers 1–16 were recorded in a randomization list held exclusively by the UMCG pharmacist.

Participants were enrolled sequentially within each group by a blinded researcher, and were assigned the next available study identification number in chronological order of inclusion. If a participant discontinued treatment within the first four weeks, a new participant was enrolled as a replacement, irrespective of the reason for dropout. As predefined in the study protocol prior to trial initiation, the replacement participant received the same study identification number and corresponding treatment allocation as the participant who discontinued. This procedure ensured that the original randomization scheme, allocation ratio, and blinding were preserved throughout the study. The replacement participant followed the entire study trajectory and all related study procedures.

Participants who discontinued treatment were not followed further and therefore could not be included in an intention-to-treat analysis. The primary analyses were performed on the per-protocol population, defined as all participants, including the replacement participants, who completed the study trajectory. In addition, sensitivity analyses were performed excluding both dropout participants and their replacements to evaluate the robustness of the findings.

Creatine and placebo solutions were prepared and dispensed by the UMCG pharmacy and were visually indistinguishable, ensuring blinding of medical staff, researchers, and participants. Laboratory analyses of creatine and guanidinoacetate were performed only after completion of the study, and all other study data were stored in a locked file until finalization. All participants, healthcare providers, and research personnel remained blinded throughout the study. Unblinding was performed only after all participants had completed their study trajectory, when the UMCG pharmacist provided the treatment allocation list to one of the researchers.

### 2.4 Study trajectory

The study trajectory for each participant consisted of a 6-week treatment period with creatine or placebo, followed by a 2-week wash-out period. Study visits were conducted at baseline, 3 weeks, 6 weeks and 8 weeks, at regular mid-week hemodialysis sessions (or for patients dialyzing twice weekly the dialysis session after the shortest interdialytic interval) (S1 Fig).

### 2.5 Intervention

Creatine monohydrate was dissolved in sterile water to create a sterile 50 mmol/L concentrate, followed by aseptic filtration. The creatine concentrate and placebo (sterile water) were filled in identical ethylene vinyl acetate 1 liter infusion bags. Details on the production and stability tests of the creatine concentrate are described in Supplementary text 1 and 2, respectively, and shown in S2 Fig.

During each dialysis session within the 6-week treatment period, the 50 mmol/L creatine concentrate or placebo was added to the dialysate to the afferent side of the extracorporeal circuit (before the dialyzer), using an infusion pump. The infusion pump flow was adjusted to the dialysate flow in order to reach the creatine concentration in the dialysate as specified by the randomization block, with creatine concentrations of 0.5 mmol/L, 1.0 mmol/L, 1.5 mmol/L and 2.0 mmol/L in the dialysate, respectively. To reach the participants’ assigned dry weight (body mass directly after dialysis), ultrafiltration volume was adjusted to account for the added volume. All other treatment characteristics like dialysate flow (500 ml/min), effective blood flow (range 150–350 ml/min), dialysis duration (3–4 hours), dialysate composition and temperature (36.0 or 36.5^◦^C) remained unchanged from the participants’ regular settings. All patients received conventional hemodialysis, no hemodiafiltration was performed in any of the study participants.

### 2.6 Biomaterial sampling and storage

At each study visit, blood was drawn before connection to the dialysis circuit and directly after the dialysis session, for storage of EDTA-anticoagulated whole blood and plasma (from lithium-heparin-anticoagulated samples, centrifuged at 2000g for 10 minutes). During hemodialysis sessions performed at study visits, the complete dialysate was collected in a 200 L tank directly connected to the dialyzer outlet. The total dialysate volume was determined gravimetrically by subtracting the tank weight before dialysis from that after dialysis. The collected dialysate was subsequently homogenized, and aliquots were stored for laboratory analysis. Participants with residual diuresis collected their complete interdialytic urine production preceding every study visit. After homogenizing and measuring the volume of the urine collection, urine samples were centrifuged (2000g, 10 minutes) and aliquoted. All samples were stored in aliquots at −80˚C until further analysis.

### 2.7 Laboratory measurements

Creatinine in plasma and dialysate was measured according to clinical routine methods, using a Roche Cobas (Roche Diagnostics, Oslo, Norway). Creatine and guanidinoacetate concentrations in dialysate, urine, plasma and whole blood (hemolyzed by freezing at −80˚C) and creatinine in urine were measured with a liquid chromatography mass spectrometry (LC-MS/MS) method, validated according to ISO15189 guidelines; using a LC30AD UPLC (Shimadzu, Kyoto, Japan) and an API-4500 mass spectrometer (SCIEX, Framingham, MA, USA). Further details are provided in Supplementary text 3.

Intra-erythrocytic creatine and guanidinoacetate concentrations were calculated as:


$$\begin{array}{c} \text{Intra}-\text{erythrocytic creatine }\left(\frac{\mu \text{mol}}{\text{L}}\right)=\frac{\text{whole blood concentration }\left(\frac{\mu \text{mol}}{\text{L}}\right)-(\text{1}-\text{hematocrit})*\text{plasma creatine}\left(\frac{\mu \text{mol}}{\text{L}}\right)}{\text{hematocrit}} \end{array}$$

Three missing hematocrit values were imputed with the average value of the available hematocrit measurements of the individual within the study period, to enable calculations of intra-erythrocytic concentrations.

### 2.8 Primary outcomes

Primary outcomes were the effect of the intervention on the difference between baseline, untreated pre-dialytic intra-erythrocytic creatine concentrations and plasma creatine concentrations, and pre-dialytic intra-erythrocytic creatine concentrations and plasma creatine concentrations after 3 and 6 weeks of treatment, compared to placebo treatment. Intra-erythrocytic creatine concentration was used as a minimally invasive marker for tissue uptake of creatine, since erythrocytes, like muscle cells, also have creatine transporters in their cell membranes [18,19].

### 2.9 Secondary outcomes

Secondary outcomes were the effect of the intervention at 6 weeks after baseline on hand grip strength (HGS); all components of the Short Physical Performance Battery (SPPB); body composition measured with BIA analyses; forced expiratory volume in 1 second; hand dexterity, assessed with a 9-hole peg test; and self-reported HRQoL, assessed with the 36-Item Short Form Health Survey (SF-36). Methods for secondary outcome assessment are described in Supplementary text 4.

BIA analyses were performed using the InBody S10 Body Composition Analyzer (inBody Co. Ltd., Seoul, Korea) before the hemodialysis session, with the participant in a supine position with arms and legs abducted from the body. Measurements were performed under standardized conditions and complemented by routine clinical assessments, including pre- and post-dialysis body weight, blood pressure, and clinical evaluation of fluid status by the dialysis nursing staff.

### 2.10 Exploratory outcomes

Exploratory outcomes were the effect of the intervention at 3 and 6 weeks after baseline on intra-erythrocytic and plasma guanidinoacetate concentrations, urinary and intradialytic creatinine excretion and estimated bodily creatine pool; direct change in plasma creatine concentration (post- minus pre-dialysis concentration); and creatine uptake. Total bodily creatine pool (based on a daily degradation rate from creatine into creatinine of 1.7% [6] was calculated as:


$$\begin{array}{c} \text{Estimated creatine pool }(\text{g})=\frac{\begin{array}{c} \left(\text{interdialytic urinary creatine excretion }(\text{g})+\text{dialytic creatinine excretion }(\text{g})\right)\\ {}*\text{ N dialysis sessions per week} \end{array}}{\text{0}.\text{017}*\text{7}} \end{array}$$

Creatine uptake was calculated as:


$$\begin{array}{c} \begin{array}{c} \text{Creatine uptake }(\text{g})=\text{ creatine concentration of the dosage }\left(\frac{\text{g}}{\text{L}}\right)*\text{dialysate volume }(\text{L})\;\mathrm{\backslash vspace}1.0mm\\ -\text{ creatine concentration in dialysate }(\text{g}) \end{array} \end{array}$$

### 2.11 Assessment of harms

Adverse events were recorded from initiation to end of the study trajectory (i.e., baseline until the end of week 8). An adverse event was defined as any new or worsening unfavorable medical occurrence arising during the study period.

Harms were assessed by systematic routine care measurements, including blood work and vital parameters, and by reporting of any adverse events by clinical personnel and participants at every dialysis session. Adverse events were recorded by the researcher and were immediately discussed with the treating nephrologist, to assess severity of the adverse event and potential relatedness to the intervention.

All adverse events were reported per complaint and per patient. Timing, frequency, severity (mild, moderate, severe), relatedness to the intervention (unlikely, plausible, likely), reasoning and consequences for the study were reported.

### 2.12 Statistical analyses

Statistical analyses were performed using IBM SPSS Statistics (version 28.0.1.0) and RStudio (version 4.4.1).

Baseline pre-dialytic intra-erythrocytic and plasma concentrations were visualized per treatment group using raincloud plots, combining a half-eye density estimate (cloud), a boxplot, and individual data points (rain) for each group. An additional cloud and boxplot representing all participants combined are included for reference using the ggplot2 and ggdist packages in RStudio.

For all variables of interest, the 3- and 6-week delta values were calculated compared to baseline. Delta values were used as the dependent variable in repeated measurement analyses using linear mixed models (MIXED procedure in SPSS) to determine the effect of creatine dosages on primary, secondary and exploratory outcomes. Time (categorical: 3 weeks and 6 weeks), treatment group, and their interaction were implemented as fixed effects. A diagonal residual covariance structure was specified for repeated measures within subjects, allowing heterogeneous variances at each time point and assuming independence of residuals within subjects. This structure was considered appropriate given that change scores from baseline largely remove within-subject correlation, and with only two post-baseline time points and 3–4 participants per group, more complex structures cannot be estimated reliably. Degrees of freedom were calculated using the Satterthwaite method. Missing data were handled within the mixed-model framework under the missing-at-random (MAR) assumption, which allows inclusion of all available observations without imputation. Analyses were performed using restricted maximum likelihood (REML) estimation. Post-hoc pairwise comparisons between treatment groups were performed using Fisher’s Least Significant Difference (LSD) method without adjustment for multiple comparisons, given the exploratory and dose-finding nature of this pilot study. Outcomes were presented as estimated marginal means and efficacy (estimated marginal mean differences between treatment groups and placebo). P-values <0.05 were considered statistically significant. However, as this study was designed as a proof-of-concept and dose-finding study, no formal power calculation was performed and the analyses were not designed to establish statistical significance. Results are therefore primarily interpreted based on the direction and magnitude of effects and their 95% confidence intervals, which inform the design of a future, adequately powered randomized controlled trial.

To determine the effect of all creatine dosages on creatine uptake and rise in plasma creatine concentration per dialysis session, linear mixed models were performed with only treatment group as fixed effect.

Sensitivity analyses were performed to test robustness of the findings. In a first set of sensitivity analyses, analyses for primary outcomes were performed with adjustment for age and sex. In a second set of sensitivity analyses, analyses for primary outcomes were performed without replacement of dropouts. In a third set of sensitivity analyses, analyses for primary outcomes were performed with different poolings of creatine concentration groups versus placebo: low dose creatine (0.5 and 1.0 mmol/L combined) and high dose creatine (1.5 and 2.0 mmol/L combined) versus placebo, and the three highest dosages of creatine (1.0, 1.5 and 2.0 mmol/L combined) versus placebo.

### 2.13 Deviations from trial registry and study protocol

#### 2.13.1 Deviations from trial registry.

Two deviations from the initial trial registry were made prior to the start of the study and were documented in the study protocol.

First, the inclusion criterion “Conventional hemodialysis, thrice weekly treatment with three to five hours per dialysis treatment” was revised to “Hemodialysis treatment in the UMCG or DCG.” This change was made to allow inclusion of patients dialyzing twice per week, as the number of eligible candidates under the original criterion was insufficient for adequate recruitment.

Second, the inclusion criterion “Hemoglobin at previous routine monthly assessment greater than or equal to 6.5 mmol/L” was replaced by the exclusion criterion “Hemoglobin at previous three routine monthly assessments on average lower than 5.5 mmol/L.” This revision was motivated by the observation that hemoglobin levels below 6.5 mmol/L are common in hemodialysis patients, and a clinically meaningful decline in hemoglobin attributable to study procedures was not anticipated. To account for natural biological variability and potential fluctuations related to fluid retention, the average hemoglobin level across the three routine assessments preceding inclusion was used as the reference value.

#### 2.13.2 Deviations from study protocol.

Two deviations from the study protocol occurred during the course of the study.

First, although the protocol specified that intra-erythrocytic creatine would be measured both before and after dialysis, post-dialysis values could not be calculated due to missing hematocrit measurements after dialysis. Pre-dialysis intra-erythrocytic creatine values were available and used in analyses as planned.

Second, cognitive testing was performed throughout the study as described in the protocol. However, due to a technical error, a substantial proportion of test results were not stored correctly and could therefore not be retrieved or used in analyses.

## 3. Results

### 3.1 Participant recruitment

**Fig 1:**
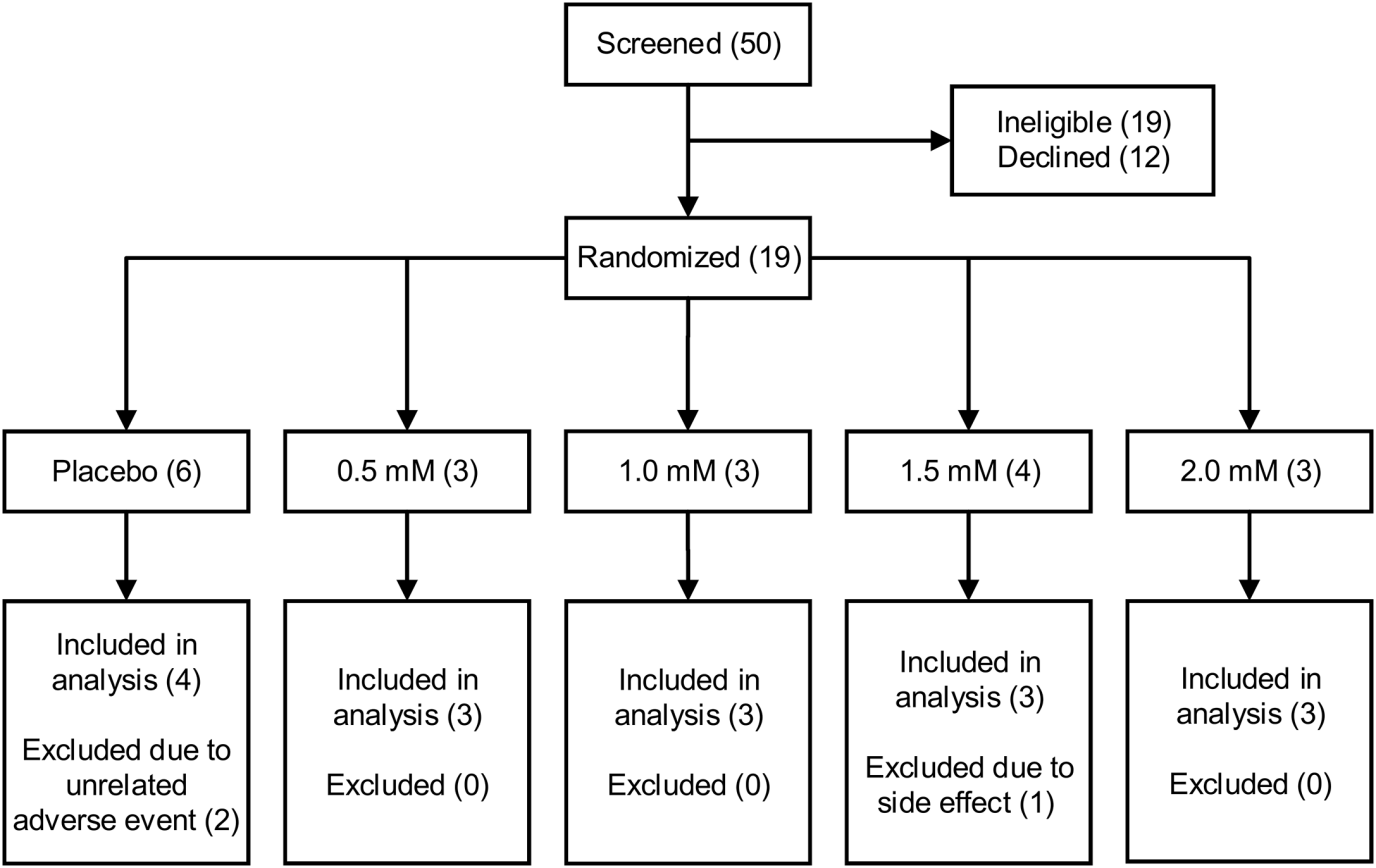
CONSORT flow diagram. Schematic overview of patient flow in the study.

Participants were recruited between January 5^th^, 2023 and January 11^th^, 2024 and the study was conducted between August 3^rd^, 2023 and March 21^th^, 2024. The study concluded after all participants completed their study trajectory. A total of 19 participants were enrolled, with 16 completing the trial (see Fig 1).

### 3.2 Baseline characteristics

**Fig 2:**
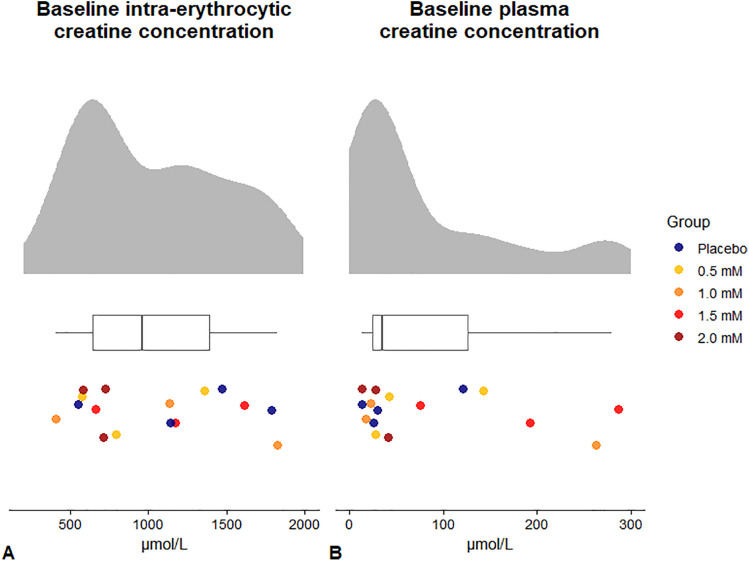
Baseline creatine concentrations. Raincloud plots showing the baseline intra-erythrocytic (A) and plasma (B) creatine concentrations for the total study population. Individual data points (rain) are shown for each participant and colored by treatment group. The boxplot displays the median, interquartile range, and whiskers extending to 1.5 times the interquartile range. The cloud represents a kernel density estimate of the data distribution.

Baseline characteristics of the 16 participants who finalized the study are shown in Table 1. Nine of the participants were female and age ranged from 27 to 79 years. The duration of hemodialysis sessions ranged from 3.0 to 4.0 hours per treatment (six patients: 3.0 hours; three patients: 3.5 hours; seven patients: 4.0 hours). Median (minimum-maximum) baseline pre-dialytic intra-erythrocytic and plasma creatine concentrations were 963 (407–1822) µmol/L and 35 (13–287) µmol/L, respectively. One patient in the 1.5 mmol/L group missed visit 4 after washout due to logistical reasons. Baseline intra-dialytic and plasma creatine concentrations are presented raincloud plots in Fig 2.

**Table 1:** Baseline characteristics of placebo and creatine treated patients.

	Placebo	Creatine	P-value
	*n =* 4	*n =* 12	
Female, *n* (%)	3 (75)	7 (58)	0.59
Age, years	52 (27, 65)	68.5 (35, 79)	0.09
Height, cm	167 (158, 186)	172 (152, 185)	0.59
Dry weight*, kg	76 (71, 131)	70 (53, 102)	0.21
BMI, kg/m^2^	30 (24, 38)	26 (21, 32)	0.21
Underlying kidney disease, *n*			0.64
Hypertension	0	2	
Diabetes mellitus	0	3	
ADPKD	1	1	
Post renal/urologic	0	1	
Glomerulonephritis	1	3	
Interstitial nephritis	0	1	
Unknown	2	1	
Plasma creatinine, µmol/L	653 (400, 953)	634 (363, 1275)	0.86
Hemoglobin, g/dL	11.8 (11.4, 13.0)	11.0 (9.3, 13.0)	0.10
Hematocrit, L/L	0.36 (0.34, 0.40)	0.33 (0.28, 0.40)	0.16
Intra-erythrocytic creatine concentration, µmol/L	1306 (550, 1789)	758 (406, 1822)	0.50
Plasma creatine concentration, µmol/L	27 (13, 121)	42 (13, 287)	0.36
Estimated creatine pool, g	63.7 (36.4, 112.8)	54.5 (31.5, 94.5)	0.85
Plasma guanidinoacetate concentration, µmol/L	1.7 (1.2, 1.8)	2.2 (1.0, 3.5)	0.30
Intra-erythrocytic guanidinoacetate concentration, µmol/L	1.7 (1.4, 2.3)	1.9 (1.0, 3.7)	0.50

For all continuous variables, median (minimum, maximum) values are presented. Between-group differences were tested using the Student’s t-test for normally distributed variables, the Mann–Whitney U test for skewed variables, and Fisher’s exact test for categorical variables.

* Dry weight refers to the body mass of the participant directly after dialysis.

### 3.3 Primary outcomes

**Fig 3:**
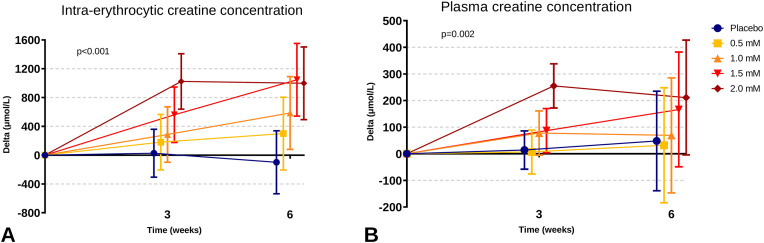
Primary outcomes. The effect of different dosages of intradialytic creatine suppletion and placebo on primary study outcomes (A. Intra-erythrocytic creatine concentration and B. Plasma creatine concentration) at three and six weeks of receiving the study intervention compared to baseline. The graphs show estimated marginal means derived from linear mixed model analyses with whiskers representing 95% confidence intervals.

At 6 weeks, a pattern consistent with a dose-dependent effect of the treatment on intra-erythrocytic creatine concentration was observed, with changes in concentration compared to placebo (95% CI) of +399 (−269, + 1066) (p = 0.22), + 685 (+17, + 1352) (p = 0.045), + 1146 (+431, + 1766) (p = 0.003) and +1098 (+431, + 1766) (p = 0.004) µmol/L for the increasing dosage treatment groups respectively (overall treatment effect p < 0.001, Fig 3A, S1 Table).

At 6 weeks, a significant overall treatment effect on plasma creatine concentration was observed (p = 0.002), with numerically increasing changes compared to placebo (95% CI) across increasing dosage treatment groups of −16 (−301, + 270) (p = 0.91), + 21 (−264, + 307) (p = 0.87), + 119 (−264, + 307) (p = 0.38) and +163 (−122, + 449) (p = 0.23) µmol/L, respectively, though none of the individual group comparisons reached statistical significance (Fig 3B, S1 Table).

Given the small sample size per dose group, individual estimates should be interpreted with caution and primarily serve to characterize the direction and magnitude of effects rather than to establish statistical significance.

Sensitivity analyses with adjustment for age and sex did not materially change the findings of the primary outcomes (S2 Table). Sensitivity analyses in which dropouts were not replaced altered the trend in plasma creatine results in the 1.5 mmol/L creatine group, as the participant with the largest increase in plasma creatine at 6 weeks was excluded from these analyses (S3 Table). Sensitivity analyses with different poolings of creatine concentrations did not materially change the findings of the primary outcomes (S4-S6 Tables).

### 3.4 Secondary outcomes

No effects of the intervention on secondary outcome parameters were observed. All secondary outcomes are presented in the S5 Text and S7 Table.

### 3.5 Exploratory outcomes

There were positive patterns consistent with dose-dependent effects of the intervention on creatine uptake per dialysis session (overall treatment effect p < 0.001) and on the elevation in plasma creatine concentration after dialysis (overall treatment effect p < 0.001, S3 Fig).

At 6 weeks, there was a positive effect of creatine supplementation on the estimated creatine pool (overall treatment effect p = 0.02, due to missing data there were no estimates on the effect in the 1.5 and the 2.0 mmol/L group) and on dialytic creatinine excretion (overall treatment effect p < 0.001). Plasma guanidinoacetate decreased numerically in patients receiving creatine, although not statistically significant. These results are shown in S8 Table, in S4 and S5 Figs and described in more detail in S6 Text.

In most participants treated with creatine, intra-erythrocytic creatine, plasma creatine, creatine pool and dialytic creatinine excretion decreased substantially, and intra-erythrocytic and plasma guanidinoacetate concentrations increased after washout (see S9 Table).

### 3.6 Adverse events and potential side effects

Out of 19 participants enrolled in the study, 4 participants that were enrolled in the placebo group (66% of total enrollments in the placebo group), and 6 that were participants enrolled in the creatine group (46% of total enrollments in the creatine group) experienced an adverse event, of which none were serious. A tabulated listing of all adverse events is shown in S10 Table.

Two adverse events, were considered likely to be a side effect of the study treatment. One participant, who received 1.5 mmol/L creatine supplementation, experienced gastrointestinal discomfort and altered taste perception during and shortly after each dialysis session with creatine treatment. The symptoms occurred three times and were self-limiting. After three dialysis sessions with creatine treatment, the participant discontinued study participation due to the burden of these complaints. The complaints resolved completely after discontinuation of the study treatment. Another participant, in the 2.0 mmol/L creatine group, experienced mild gastrointestinal discomfort during and shortly after each dialysis session with creatine treatment. The symptoms occurred during each dialysis session during the 6 week treatment period and were self-limiting. Due to the mild nature of the complaints, this participant agreed to complete the study. The complaints resolved completely after completion of the study treatment. Given the consistent temporal association between initiation of the intervention and onset of the symptom in these two participants, nausea and dysgeusia were considered potential side effects of the treatment.

Two patients in the placebo group experienced adverse events and dropped out of the study. However, these dropouts were unrelated to creatine supplementation (see Fig 1).

## 4. Discussion

This randomized placebo-controlled pilot study showed that intradialytic creatine supplementation with continuous concentrations ranging from 0.5 to 2.0 mmol/L in patients treated with hemodialysis, is feasible, safe and well tolerated in the majority of the patients. The observed increases in intra-erythrocytic and plasma creatine concentrations provide a first indication of the direction and order of magnitude of the treatment effect, and support the rationale for a larger randomized controlled trial.

Our study demonstrates that intradialytic creatine supplementation results in effective, uptake of creatine from the dialysate, in a pattern consistent with a dose-dependent effect. In contrast, the placebo group experienced a loss of creatine into the dialysate, consistent with the concentration gradient from plasma to the dialysate, and in line with existing evidence [4,13].

Over the 6-week treatment period, pre-dialytic intra-erythrocytic and plasma creatine levels increased in most participants, accompanied by an increase in the bodily creatine pool, demonstrating the feasibility and effectiveness of intradialytic creatine supplementation for long term creatine status improvement. Dosages of 1.0 mmol/L to 2.0 mmol/L appeared to be effective, with no material differences at 6 weeks between the 1.5 and 2.0 mmol/L dosage treatment groups, indicating there might be a ceiling effect.

Plasma guanidinoacetate decreased in patients receiving creatine, aligning with findings from a previous randomized controlled trial (RCT) with oral creatine supplementation [20]. This likely reflects GATM downregulation, indicating downregulation of endogenous creatine synthesis [21]. Furthermore, creatine uptake per dialysis session decreased numerically over time, suggesting a gradual saturation of the creatine pool, resulting in declining uptake capacity and endogenous synthesis. We speculate that with stable creatine dosing over time, the daily 1.7% conversion into creatinine will eventually balance out with supplementation and reach a steady state. Further research is needed to determine when this plateau occurs, considering potential feedback systems on the endogenous synthesis rate.

Interestingly, baseline intra-erythrocytic and plasma creatine levels in our study were not lower compared to other populations [22,23]. For example, a general population study from Jiao et al. found intra-erythrocytic creatine concentrations of 409 ± 85 µmol/L [22], whereas we found concentrations ranging from 406–1822 µmol/L in patients treated with hemodialysis. The use of different laboratory methods may partially explain these differences, with Jiao et al. using an enzymatic assay, whereas we used an LC-MS based assay. Furthermore, besides being influenced by intra-cellular creatine uptake through the creatine transporters in the cell membranes [18,19], intra-erythrocytic creatine concentrations are also influenced by erythrocyte age, with younger erythrocytes having higher intracellular creatine concentrations [24]. Intra-erythrocytic creatine has previously been linked to markers of erythrocyte turnover [25]. In patients treated with hemodialysis, blood loss, dialysis induced hemolysis and erythropoietin use may lead to younger circulating erythrocytes. Nevertheless, the increase in intra-erythrocytic creatine concentration during the treatment period clearly demonstrates the cellular uptake of creatine, which was the aim of measuring intra-erythrocytic creatine in our study. In a general population study, rising intra-erythrocytic and plasma creatine concentrations after oral creatine supplementation have also been demonstrated [23].

Physical function parameters and body composition did not improve among our study participants. Notably, this study was not designed to study changes in these parameters, with a treatment period of 6 weeks likely being too short to detect clinically relevant improvements. We included these measurements to obtain pilot data, facilitating the design for a future larger RCT, in which physical test results and HRQoL will be main outcome parameters. In an earlier RCT, in which patients treated with hemodialysis were orally supplemented with 20 gram doses of creatine monohydrate daily during a 1-week loading phase, followed by 5 grams daily for 3 weeks [15], and in a follow-up study in patients treated with hemodialysis, with oral supplementation of 5 grams of creatine monohydrate daily for 12 months, creatine supplementation significantly increased fat-free body mass compared to placebo, measured with a DXA-scan [16]. The differences with the findings in our study may be explained by a higher dose of creatine administered, longer treatment period (in the follow-up study) and a more sensitive body composition measurement method (DXA compared to BIA) [15,16]. Even at the highest dialysate concentration, the estimated creatine uptake was approximately 7 grams per dialysis session, corresponding to an average daily exposure of ~3 grams for patients dialyzed three times per week. In comparison, most supplementation studies typically include a loading phase of 20 g/day followed by a maintenance dose of 5 g/day, of which approximately 80–90% is absorbed [15,16]. Thus, systemic exposure in our trial was likely lower than in most oral regimens, which may have limited the magnitude of functional effects observed. Furthermore, recent meta-analyses indicate that handgrip strength is relatively insensitive to detect creatine-related improvements, particularly in older adults, suggesting that future studies should include more responsive performance measures [26,27].

Another recent oral creatine supplementation study in hemodialysis also showed clinical improvements and muscle mass increase measured with BIA, after only 8 weeks of supplementation [28]. This study also provided a higher dose of creatine compared to our study. It is important to note that in contrast with our study, this study was not placebo-controlled and not blinded, which may influence outcomes of the study. Furthermore, there may be a priori differences in vitality and mobility of included patients, which if present, could potentially explain differences in findings with our study. Nevertheless, the positive findings of these oral creatine supplementation studies provide promising data regarding creatine supplementation in hemodialysis. These benefits could likely be extrapolated to intradialytic creatine supplementation, with the added benefits of easy implementation in routine care, no need for additional fluid intake, therapy compliance, and potentially better tolerance regarding gastro-intestinal side-effects compared to oral intake.

Some evidence exists that creatine supplementation may increase body water [16,29], although there is no evidence for long term water retention caused by creatine [29]. Notably, in patients requiring ultrafiltration for regulating fluid balance, ultrafiltration directly affects the body water measurements post-dialysis. Nevertheless, we found no effect of creatine supplementation on intracellular or extracellular body water post-dialysis, measured with BIA, consistent with previous creatine supplementation studies in hemodialysis [15,16,28], although one patient in our study experienced fluid congestion attributed to aortic valve stenosis.

Creatine supplementation was generally well tolerated. Only one patient in the 1.5 mmol/L group was excluded because of potential side effects (nausea and dysgeusia), and another patient in the 2.0 mmol/L group experienced milder complaints of nausea and completed the study. The two dropouts in the placebo group were unrelated to creatine supplementation. There is anecdotal evidence for gastro-intestinal complaints as a result of oral creatine supplementation [30–32]. In a study with patients treated with hemodialysis receiving oral creatine supplementation, no side effects occurred [15,28]. To our knowledge, no existing literature addresses side effects of creatine supplementation in humans when administered through routes other than orally. Most importantly, we observed no serious adverse events, suggesting that intradialytic creatine supplementation is safe.

Strengths of this study include the randomized, placebo controlled, double-blinded study design, and the many comprehensive measurements of creatine, creatinine and guanidinoacetate in a variety of biological materials, including plasma, erythrocytes, urine and dialysate. A unique feature is the complete capture of waste dialysate, allowing for precise, quantitative measurements of substances lost to the dialysate, complemented with urine collections, as a robust equivalent for 24-hour urine collections in non-dialyzing patients. A limitation was missing data on urine collections needed to calculate the creatine pool. Furthermore, error margins in multiple components of the calculation could increase the final error margin. The stable intra-patient ratio between creatinine excreted in dialysate versus urine allowed us to observe a qualitative trend of increasing total bodily creatine through solely the dialytic creatinine excretion, although not in all treatment groups. Because this was an exploratory pilot study, no formal correction for multiple comparisons was applied. Consequently, the possibility of type I error inflation cannot be excluded, and the results should be interpreted as hypothesis-generating. In addition, the small sample size and short study duration limit the ability to draw firm conclusions regarding long-term safety and clinical efficacy. Furthermore, estimates derived from the small groups should be interpreted as descriptive rather than representative. Future larger and longer-term studies are warranted to confirm the findings of this study and to assess potential clinical benefits and long-term tolerability of intradialytic creatine supplementation.

In conclusion, this proof-of-concept pilot study demonstrates that intradialytic creatine supplementation is a feasible, safe and generally well-tolerated method for replenishing the total creatine pool in stable hemodialysis patients. The observed increases in creatine concentrations suggest that dialysate concentrations of 1.0 to 2.0 mmol/L creatine represent an effective dosage range. These findings support the rationale for a larger RCT with a longer treatment period to establish efficacy and explore the potential of intradialytic creatine supplementation to improve health-related quality of life and reduce mortality risk in this population.

## Supporting information

S1 Text Creatine concentrate production (methods). (DOCX)

S2 Text Stability studies for creatine concentrate (methods). (DOCX)

S3 Text Laboratory measurements (methods). (DOCX)

S4 Text Secondary outcome assessment (methods). (DOCX)

S5 Text Secondary outcomes (results). (DOCX)

S6 Text Exploratory outcomes (results). (DOCX)

S1 Table Primary outcomes. Results from linear mixed model analyses: The effect of the study treatment per treatment group on primary outcomes compared to placebo at 3 weeks and at 6 weeks of receiving the study intervention.
(DOCX)

S2 Table Sensitivity analysis for primary outcomes adjusted for sex and age. Results from sensitivity linear mixed model analyses with adjustments for sex and age: The effect of the study treatment per treatment group on primary outcomes compared to placebo at 3 weeks and at 6 weeks of receiving the study intervention.
(DOCX)

S3 Table Sensitivity analyses for primary outcomes without replacement of dropouts. Results from sensitivity linear mixed model analyses in which participants replacing patients who dropped out were not included in the analyses: The effect of the study treatment per treatment group on primary outcomes compared to placebo at 3 weeks and at 6 weeks of receiving the study intervention.
(DOCX)

S4 Table Sensitivity analyses for primary outcomes with all creatine groups pooled. Results from sensitivity linear mixed model analyses: The effect of the study treatment for all groups with creatine supplementation pooled into one intervention group on exploratory outcomes compared to placebo at 3 weeks and at 6 weeks of receiving the study intervention.
(DOCX)

S5 Table Sensitivity analyses for primary outcomes with three highest creatine groups pooled. Results from sensitivity linear mixed model analyses: The effect of the study treatment for the three highest dosage treatment groups of creatine supplementation pooled into one intervention group on exploratory outcomes compared to placebo at 3 weeks and at 6 weeks of receiving the study intervention.
(DOCX)

S6 Table Sensitivity analyses for primary outcomes with two lowest and two highest creatine groups pooled. Results from sensitivity linear mixed model analyses: The effect of the study treatment for the two lowest and two highest dosage treatment groups of creatine supplementation pooled into two intervention groups on primary outcomes compared to placebo at 3 weeks and at 6 weeks of receiving the study intervention.
(DOCX)

S7 Table Secondary outcomes. Results from linear mixed model analyses: The effect of the study treatment per treatment group on secondary outcomes compared to placebo at 3 weeks and at 6 weeks of receiving the study intervention.
(DOCX)

S8 Table Exploratory outcomes. Results from linear mixed model analyses: The effect of the study treatment per treatment group on exploratory outcomes compared to placebo at 3 weeks and at 6 weeks of receiving the study intervention.
(DOCX)

S9 Table Change in primary and exploratory outcomes during the 2-week washout period. (DOCX)

S10 Table Listing of all adverse events during the study period. (DOCX)

S1 Fig Schematic overview of study trajectory per participant. (DOCX)

S2 Fig Stability testing. Results of the stability tests of the investigational product, showing creatine concentrations over time for the technical batches and the confirmation batch.
(DOCX)

S3 Fig Exploratory outcomes. A. Mean creatine uptake per treatment group per study visit. B. Mean creatine uptake per kg of fat free mass per group per study visit. C. Mean change in plasma creatine concentration per treatment group per study visit. Whiskers represent minimum and maximum values and p-values are based on an overall between-group comparison.
(DOCX)

S4 Fig Exploratory outcomes. The effect of different dosages of intradialytic creatine suppletion and placebo on exploratory study outcomes (A. Estimated total creatine pool. B. Dialytic creatinine excretion. C. Plasma guanidino acetate. D. Intra-erythrocytic guanidinoacetate) at three and six weeks of receiving the study intervention compared to baseline. The graphs show estimated marginal means derived from linear mixed model analyses with whiskers representing 95% confidence intervals.
(DOCX)

S5 Fig Creatine excretion. Urinary and dialytic creatinine excretion per study visit per individual participant. Placebo treated patients are marked with a “*”. Note that in participant 2, 6, 9, 10, 11, 14, 15 and 16 some urinary excretions could not be shown due to missing urine collections. Participant 5 and 8 had no residual diuresis. A. Creatinine excretion per dialytic interval (end of prior dialysis session to end of current dialysis session). B. Mean creatinine excretion per day (creatine excretion per dialytic interval divided by height of dialytic interval in days).
(DOCX)
