# Supplementary material for: Feasibility, safety and tolerability of intradialytic creatine supplementation in hemodialysis: A double-blind, randomized, placebo-controlled pilot study
Source: PLoS One. 2026 Aug 5;21(8):e0354883. doi: 10.1371/journal.pone.0354883 (PMC13440799; doi:10.1371/journal.pone.0354883)
Supplement: Supplementary file 1 [file pone.0354883.s001.docx]

**1. Creatine concentrate production**

Creatine monohydrate, Creapure®, produced by AlzChem Trostberg, Germany, and tested to be compliant for clinical use and in line with the dialysis pharmacopoeia by Laboratory LS, was used for preparation of a 50 mmol/L creatine concentrate. This concentrate of creatine was dissolved in water for injections, followed by aseptic filtration and filling through a 0.22 µm filter (Sartorius, Göttingen, Germany) under aseptic conditions (Grade A-Laminar Air Flow hood with Grade B cleanroom as background), in order to obtain a sterile solution (Ace Pharmaceuticals, Zeewolde, The Netherlands). Placebo consisted of sterile water. The creatine concentrate and placebo were filled in identical ethylene vinyl acetate 1 liter infusion bags (Baxter Exacta Mix bags, Baxter, Utrecht, The Netherlands). Stability studies for the 50 mmol/L creatine concentrate showed that the creatine concentration is stable for at least 9 months at refrigerated temperature (5±3 ˚Celsius).
