# Supplementary material for: Feasibility, safety and tolerability of intradialytic creatine supplementation in hemodialysis: A double-blind, randomized, placebo-controlled pilot study
Source: PLoS One. 2026 Aug 5;21(8):e0354883. doi: 10.1371/journal.pone.0354883 (PMC13440799; doi:10.1371/journal.pone.0354883)
Supplement: Supplementary file 2 [file pone.0354883.s002.docx]

**2. Creatine concentrate stability studies**

To exclude decay of creatine to creatinine in storage bags, we performed stability studies on the creatine concentrate for hemodialysis (50 mmol/L) used to achieve the desired creatine concentrations in the dialysis fluid for the study. We initially performed these stability studies on the creatine concentrate manufactured by Ace Pharmaceuticals (Zeewolde, The Netherlands) using 1 liter ethylene vinyl acetate infusion bags from B. Braun (Melsungen, Germany) . All tests were performed according to Ph. Eur. and ICH Q1A(R2) guidelines. Release specifications for creatine were 95-105 % drug content and end of shelf-life specification was <90% or >110% drug content. Initially, the stability was tested on three technical batches refrigerated and ambient temperature. The results of these stability tests are shown in **Fig S2**, and showed that creatine was stable for at least 9 months at refrigerated temperature (2-8 ^◦^Celsius). At ambient temperature, the creatine concentration had dropped below the release specifications at 9 months. A confirmatory stability study was performed because of a switch in dialysis bag manufacturer from B. Braun to Baxter (Deerfield, Illinois). The results demonstrated that creatine is also stable at refrigerated temperature (2-8 degrees Celsius) in 1 liter ethylene vinyl acetate Baxter bags for at least 9 months.
