# Supplementary material for: Feasibility, safety and tolerability of intradialytic creatine supplementation in hemodialysis: A double-blind, randomized, placebo-controlled pilot study
Source: PLoS One. 2026 Aug 5;21(8):e0354883. doi: 10.1371/journal.pone.0354883 (PMC13440799; doi:10.1371/journal.pone.0354883)
Supplement: Supplementary file 3 [file pone.0354883.s003.docx]

**3. Laboratory measurements**

Guanidinoacetate, creatine and creatinine were separated using a Phenomenex Kinetex Evo™ C18 (2.6 µm, 100 Å, 150x4.6 mm) column and detected using positive-ion electrospray ionization with the following multiple reaction monitoring (MRM) transitions: m/z 132🡪90 for creatine, 118🡪76 for guanidinoacetate and 114🡪86 for creatinine. Internal standards were detected using the following MRM transitions: m/z 135🡪93 for D3-creatine, m/z 120🡪78 for 13C2-guanicinoacetate and 117🡪89 for D3-creatinine. Data was analyzed using Analyst MD 1.6.3 (Sciex).
