# Supplementary material for: Feasibility, safety and tolerability of intradialytic creatine supplementation in hemodialysis: A double-blind, randomized, placebo-controlled pilot study
Source: PLoS One. 2026 Aug 5;21(8):e0354883. doi: 10.1371/journal.pone.0354883 (PMC13440799; doi:10.1371/journal.pone.0354883)
Supplement: Supplementary file 4 [file pone.0354883.s004.docx]

**4. Secondary outcome assessment**

Hand grip strength

Hand grip strength was assessed in kilograms (kg) before dialysis, at the non-fistula arm, using the Jamar Hydraulic Hand Dynamometer (Patterson Medical JAMAR 5030J1, Warenville, Canada). The hand grip strength was assessed in seated position, with shoulders in adduction, arms rotated into neutral position, elbows flexed to 90°, and forearms, and wrists held in neutral position. Participants were instructed to perform a maximal isometric contraction, encouraged with voiced commands. Hand grip strength was tested three times with an interval of 30 seconds between each attempt. For the analyses the highest value was used.

Short Physical Performance Battery

The Short Physical Performance Battery (SPPB) consisting of three separate tests was conducted before dialysis. For each test, points are awarded according to the performance: For testing balance, participants were asked to stand still for at least 10 seconds in three progressively difficult positions, with their feet together in side-by-side, semi-tandem, and tandem positions. Lower limb strength was tested with the five times sit to stand test (FTSTS), for which participants were asked to fold their arm across their chests while standing up and sitting down five times as quickly as possible, the time will be recorded in seconds. The 4-meter Gait Speed Test (4MWT) was used to measure locomotion. Participants were asked to walk at a regular pace. The time needed to walk 4 meters was recorded in seconds.

Body composition

Body composition was determined using a multifrequency bioelectrical impedance analyzer (BIA) (InBody S10; InBody Co., Ltd., Seoul, Korea) at 5, 50, 100, and 200 Hz, which allows to distinguish between lean body mass and fat body mass. BIA measurements were performed immediately after the hemodialysis session, with the participant in supine position with arms and legs abducted from the body.

Forced Expiratory Volume

Forced expiratory volume (FEV_1_) was measured in liters, both before and after dialysis, using an Asma-1 handheld spirometer (Vitalograph, Buckingham, UK).

9-Hole Peg Test

The 9-Hole Peg Test (9-HPT), Sammsons Preston Rolyan, Chicaco, Illinois) is a measure of hand dexterity, requiring participants to place and remove nine pegs into nine holes, one at a time, as quickly as possible. The 9-HPT was performed before dialysis with both hands, starting with the dominant hand.

Self-reported physical health

The Short Form 36 Health Survey (SF36) to assess HRQoL was administered during the visit 1, 3 and 4.
