# Supplementary material for: Feasibility, safety and tolerability of intradialytic creatine supplementation in hemodialysis: A double-blind, randomized, placebo-controlled pilot study
Source: PLoS One. 2026 Aug 5;21(8):e0354883. doi: 10.1371/journal.pone.0354883 (PMC13440799; doi:10.1371/journal.pone.0354883)
Supplement: Supplementary file 5 [file pone.0354883.s005.docx]

**5. Secondary outcomes**

Changes in the Physical Component Score and the Mental Component Score, as markers for HRQoL derived from the SF-36 questionnaire, were not different between placebo and different dosage groups (p=0.47 and p=0.62, respectively). All secondary outcomes assessed with linear mixed model analyses are presented in **Table S1**.
