# Supplementary material for: Feasibility, safety and tolerability of intradialytic creatine supplementation in hemodialysis: A double-blind, randomized, placebo-controlled pilot study
Source: PLoS One. 2026 Aug 5;21(8):e0354883. doi: 10.1371/journal.pone.0354883 (PMC13440799; doi:10.1371/journal.pone.0354883)
Supplement: Supplementary file 6 [file pone.0354883.s006.docx]

**6. Exploratory outcomes**

The creatine uptake during a dialysis session compared to placebo (95% CI) was +13.6 (+3.1, +24.2) (p=0.01), +29.8 (+19.2, +40.4) (p<0.001), +33.3 (+22.7, +43.9) (p<0.001) and +44.2 (+33.6, +54.8) (p<0.001) mmol/L for increasing dosage groups, respectively (overall treatment effect p<0.001), as shown in **Fig S3A**. Identical associations were observed when creatine uptake was calculated per kg of fat free mass, as shown in **Fig S3B**. Creatine uptake per dialysis session decreased over time in all treatment groups. In the placebo group the median (min;max) loss of creatine per dialysis session was -0.65(-6.0;-0.4) mmol/L.

The rise in plasma creatine concentration during a dialysis session (concentration post- minus concentration pre-dialysis) compared to placebo (95% CI) was +204 (+106, +301) (p<0.001), +383 (+285, +480) (p<0.001), +528 (+430, +626) (p<0.001), and +660 (+563, +758) (p<0.001) mmol/L for increasing dosage groups, respectively (overall treatment effect p<0.001), as shown in **Fig S3C**. In the washout period, a small creatine loss during dialysis was observed in all groups.

There was a positive effect of creatine supplementation on the estimated creatine pool (p=0.02), as shown in **Table S5** and **Fig S3A**. Unfortunately, there were missing data on urinary creatinine excretion in the 1.5 and the 2.0 mmol/L group, which were needed for estimation of the creatine pool. However, in 10 out of 11 creatine-treated participants with at least two available datapoints within the 6 week treatment period, an increase in the creatine pool compared to baseline was observed, compared with 1 out of 2 placebo treated participants with available data.
In participants with residual diuresis, urinary creatinine excretion was on average 41% (range 3% to 65%) of the total (dialysate and urine) creatinine excretion. This ratio was consistent within individuals between observations, as can be seen in **Fig S5**. Therefore, creatinine excretion in the dialysate could serve as a proxy to investigate trends over time in total creatinine excretion/creatine pool. Dialytic creatinine excretion was increased by the intervention (p<0.001), as shown in **Table S5** and **Fig S3B**).

We observed a numerical decline in pre-dialytic plasma guanidinoacetate concentrations over the 6-week treatment period (p=0.37), as shown in **Table S5** and **Fig S3C**. For pre-dialytic intra-erythrocytic guanidinoacetate levels, a dose-independent difference between groups was observed (p=0.03, as shown in **Table S5** and **Fig S3D**.

After washout, in most participants intra-erythrocytic creatine, plasma creatine, creatine pool and dialytic creatinine excretion decreased substantially, and in most participants there was an increase in intra-erythrocytic and plasma guanidinoacetate concentrations. Changes of outcome parameters in the wash-out period are shown in **Table S6**.
