# Supplementary material for: Feasibility, safety and tolerability of intradialytic creatine supplementation in hemodialysis: A double-blind, randomized, placebo-controlled pilot study
Source: PLoS One. 2026 Aug 5;21(8):e0354883. doi: 10.1371/journal.pone.0354883 (PMC13440799; doi:10.1371/journal.pone.0354883)
Supplement: Supplementary file 7 [file pone.0354883.s007.docx]

| **S1 Table**. **Primary outcomes.** Results from linear mixed model analyses: The effect of the study treatment per treatment group on primary outcomes compared to placebo at 3 weeks and at 6 weeks of receiving the study intervention. | | | | | | | | | | | | | | | | | | | | | |
| --- | --- | --- | --- | --- | --- | --- | --- | --- | --- | --- | --- | --- | --- | --- | --- | --- | --- | --- | --- | --- | --- |
| Delta over time: | df |  | Placebo |  | 0.5 mmol/L | |  |  | 1.0 mmol/L | |  |  | 1.5 mmol/L | |  |  | 2.0 mmol/L | |  |  | Overall |
|  |  |  | mean |  | mean | efficacy* | p-value |  | mean | efficacy* | p-value |  | mean | efficacy* | p-value |  | mean | efficacy* | p-value |  | p-value |
| Intra-erythrocytic creatine (µmol/L) |  |  |  |  |  |  |  |  |  |  |  |  |  |  |  |  |  |  |  |  |  |
| 3 weeks | 11 |  | 26  (-307, 359) |  | 180  (-204, 565) | 154  (-354, 663) | 0.52 |  | 286  (-98, 671) | 261  (-248, 769) | 0.28 |  | 560  (175, 945) | 534  (26, 1043) | 0.04 |  | 1023  (639, 1408) | 997  (489, 1506) | 0.001 |  | <0.001 |
| 6 weeks | 11 |  | -100  (-537, 337) |  | 299  (-206, 804) | 399  (-269, 1066) | 0.22 |  | 585  (80, 1090) | 685  (17, 1352) | 0.045 |  | 1046  (542, 1551) | 1146  (431, 1766) | 0.003 |  | 999  (494, 1503) | 1098  (431, 1766) | 0.004 |  |  |
|  |  |  |  |  |  |  |  |  |  |  |  |  |  |  |  |  |  |  |  |  |  |
| Plasma creatine (µmol/L) |  |  |  |  |  |  |  |  |  |  |  |  |  |  |  |  |  |  |  |  |  |
| 3 weeks | 11 |  | 14  (-58, 86) |  | 7  (-76, 89) | -8 (-117, 102) | 0.88 |  | 78  (-5, 161) | 64  (-46, 173) | 0.23 |  | 87  (4, 170) | 73  (-36, 183) | 0.17 |  | 255  (172, 338) | 241  (131, 350) | 0.001 |  | 0.002 |
| 6 weeks | 11 |  | 48  (-139, 235) |  | 32  (-184, 248) | -16  (-301, 270) | 0.91 |  | 69  (-147, 285) | 21  (-264, 307) | 0.87 |  | 167  (-49, 382) | 119  (-264, 307) | 0.38 |  | 211  (-4, 427) | 163  (-122, 449) | 0.23 |  |  |
| *Efficacy: Difference compared to placebo | | | | | | | | | | | | | | | | | | | | | |
