# Supplementary material for: Feasibility, safety and tolerability of intradialytic creatine supplementation in hemodialysis: A double-blind, randomized, placebo-controlled pilot study
Source: PLoS One. 2026 Aug 5;21(8):e0354883. doi: 10.1371/journal.pone.0354883 (PMC13440799; doi:10.1371/journal.pone.0354883)
Supplement: Supplementary file 8 [file pone.0354883.s008.docx]

| **S2 Table. Sensitivity analysis for primary outcomes adjusted for sex and age.** Results from sensitivity linear mixed model analyses with adjustments for sex and age: The effect of the study treatment per treatment group on primary outcomes compared to placebo at 3 weeks and at 6 weeks of receiving the study intervention. | | | | | | | | | | | | | | | | | | | | | |
| --- | --- | --- | --- | --- | --- | --- | --- | --- | --- | --- | --- | --- | --- | --- | --- | --- | --- | --- | --- | --- | --- |
| Delta over time: | df |  | Placebo |  | 0.5 mmol/L | |  |  | 1.0 mmol/L | |  |  | 1.5 mmol/L | |  |  | 2.0 mmol/L | |  |  | Overall |
|  |  |  | mean |  | mean | efficacy* | p-value |  | mean | efficacy* | p-value |  | mean | efficacy* | p-value |  | mean | efficacy* | p-value |  | p-value |
| Intra-erythrocytic creatine (µmol/L) |  |  |  |  |  |  |  |  |  |  |  |  |  |  |  |  |  |  |  |  |  |
| 3 weeks | 11 |  | 154  (-310,  -618) |  | 135  (-309, 579) | -19  (-667, 630) | 0.95 |  | 275  (-171, 722) | 121  (-534, 777) | 0.68 |  | 552  (106, 998) | 398  (-256, 1053) | 0.21 |  | 980  (534, 1424) | 825  (178, 1474) | 0.02 |  | <0.001 |
| 6 weeks | 11 |  | -16  (-452,  -320) |  | 254  (-238, 746) | 270  (-393, 934) | 0.39 |  | 574  (79, 1068) | 589  (-74, 1254) | 0.08 |  | 945  (345, 1545) | 961  (220, 1702) | 0.02 |  | 955  (463, 1447) | 971  (308, 1635) | 0.009 |  |  |
|  |  |  |  |  |  |  |  |  |  |  |  |  |  |  |  |  |  |  |  |  |  |
| Plasma creatine (µmol/L) |  |  |  |  |  |  |  |  |  |  |  |  |  |  |  |  |  |  |  |  |  |
| 3 weeks | 11 |  | 24  (-63, 110) |  | 4  (-91, 99) | -20  (-152, 112) | 0.74 |  | 74  (-22, 168) | 50  (-81, 181) | 0.42 |  | 83  (-12, 178) | 59  (-71, 190) | 0.33 |  | 252  (157, 347) | 228  (97, 360) | 0.003 |  | 0.01 |
| 6 weeks | 11 |  | 58  (-128, 243) |  | 30  (-184, 242) | -28  (-311, 255) | 0.83 |  | 65  (-148, 278) | 7  (-276, 290) | 0.43 |  | 163  (-50, 376) | 105  (-178, 388) | 0.26 |  | 209 (-4, 422) | 151  (-132, 435) | 0.74 |  |  |
| *Efficacy: Difference compared to placebo | | | | | | | | | | | | | | | | | | | | | |
