# Supplementary material for: Feasibility, safety and tolerability of intradialytic creatine supplementation in hemodialysis: A double-blind, randomized, placebo-controlled pilot study
Source: PLoS One. 2026 Aug 5;21(8):e0354883. doi: 10.1371/journal.pone.0354883 (PMC13440799; doi:10.1371/journal.pone.0354883)
Supplement: Supplementary file 9 [file pone.0354883.s009.docx]

| **S3** **Table. Sensitivity analyses for primary outcomes without replacement of dropouts.** Results from sensitivity linear mixed model analyses in which participants replacing patients who dropped out were not included in the analyses: The effect of the study treatment per treatment group on primary outcomes compared to placebo at 3 weeks and at 6 weeks of receiving the study intervention. | | | | | | | | | | | | | | | | | | | | | |
| --- | --- | --- | --- | --- | --- | --- | --- | --- | --- | --- | --- | --- | --- | --- | --- | --- | --- | --- | --- | --- | --- |
| Delta over time: | df |  | Placebo |  | 0.5 mmol/L | |  |  | 1.0 mmol/L | |  |  | 1.5 mmol/L | |  |  | 2.0 mmol/L | |  |  | Overall |
|  |  |  | mean |  | mean | efficacy* | p-value |  | mean | efficacy* | p-value |  | mean | efficacy* | p-value |  | mean | efficacy* | p-value |  | p-value |
| Intra-erythrocytic creatine (µmol/L) |  |  |  |  |  |  |  |  |  |  |  |  |  |  |  |  |  |  |  |  |  |
| 3 weeks | 8 |  | 128  (-428, 683) |  | 180  (-273, 634) | 53  (-664, 770) | 0.87 |  | 286  (-167, 740) | 159  (-558, 876) |  |  | 640  (84, 1195) | 512  (-274, 1297) | 0.17 |  | 1023 (570, 1477) | 895  (179, 1612) | 0.02 |  | 0.004 |
| 6 weeks | 9 |  | -69  (-631, 493) |  | 299  (-263, 861) | 368  (-427, 1163) | 0.32 |  | 585  (23, 1147) | 654  (-140, 1449) | 0.10 |  | 948  (260, 1636) | 1017 (128, 1905) | 0.03 |  | 999  (437, 1560) | 1068 (273, 1862) | 0.01 |  |  |
|  |  |  |  |  |  |  |  |  |  |  |  |  |  |  |  |  |  |  |  |  |  |
| Plasma creatine (µmol/L) |  |  |  |  |  |  |  |  |  |  |  |  |  |  |  |  |  |  |  |  |  |
| 3 weeks | 9 |  | 18  (-72, 108) |  | 7  (-83, 96) | -11  (-138, 116) | 0.85 |  | 78  (-12, 168) | 60  (-67, 187) | 0.31 |  | 62  (-48, 171) | 44  (-98, 186) | 0.50 |  | 255  (164, 345) | 237  (110, 364) | 0.002 |  | 0.051 |
| 6 weeks | 9 |  | 64  (-133, 262) |  | 32  (-165, 230) | -32  (-211, 247) | 0.80 |  | 69  (-128, 266) | 5  (-274, 284) | 0.97 |  | 32  (-210, 274) | -37  (-344, 280) | 0.82 |  | 211 (14, 409) | 147  (-132, 426) | 0.26 |  |  |
| *Efficacy: Difference compared to placebo | | | | | | | | | | | | | | | | | | | | | |
