# Supplementary material for: Feasibility, safety and tolerability of intradialytic creatine supplementation in hemodialysis: A double-blind, randomized, placebo-controlled pilot study
Source: PLoS One. 2026 Aug 5;21(8):e0354883. doi: 10.1371/journal.pone.0354883 (PMC13440799; doi:10.1371/journal.pone.0354883)
Supplement: Supplementary file 10 [file pone.0354883.s010.docx]

| **S4 Table. Sensitivity analyses for primary outcomes with all creatine groups pooled.** Results from sensitivity linear mixed model analyses: The effect of the study treatment for all groups with creatine supplementation pooled into one intervention group on exploratory outcomes compared to placebo at 3 weeks and at 6 weeks of receiving the study intervention. | | | | | | |
| --- | --- | --- | --- | --- | --- | --- |
| Delta over time: | df | Placebo |  | Creatine |  |  |
|  |  | Mean |  | Mean | Difference to placebo | P-value |
| Intra-erythrocytic creatine (µmol/L) |  |  |  |  |  |  |
| 3 weeks | 14 | 26 (-407, 456) |  | 513 (263, 762) | 487 (-13, 986) | 0.06 |
| 6 weeks | 14 | -100 (-585, 386) |  | 732 (452, 1013) | 832 (271, 1393) | 0.01 |
|  |  |  |  |  |  |  |
| Plasma creatine (µmol/L) |  |  |  |  |  |  |
| 3 weeks | 14 | 14 (-95, 123) |  | 106 (43, 170) | 92 (-34, 219) | 0.14 |
| 6 weeks | 14 | 48 (-129, 224) |  | 120 (18, 222) | 72 (-132, 276) | 0.46 |
