# Supplementary material for: Feasibility, safety and tolerability of intradialytic creatine supplementation in hemodialysis: A double-blind, randomized, placebo-controlled pilot study
Source: PLoS One. 2026 Aug 5;21(8):e0354883. doi: 10.1371/journal.pone.0354883 (PMC13440799; doi:10.1371/journal.pone.0354883)
Supplement: Supplementary file 11 [file pone.0354883.s011.docx]

| **S5 Table. Sensitivity analyses for primary outcomes with three highest creatine groups pooled.** Results from sensitivity linear mixed model analyses: The effect of the study treatment for the three highest dosage treatment groups of creatine supplementation pooled into one intervention group on exploratory outcomes compared to placebo at 3 weeks and at 6 weeks of receiving the study intervention. | | | | | | |
| --- | --- | --- | --- | --- | --- | --- |
| Delta over time: | df | Placebo |  | 1.0 & 1.5 & 2.0 mmol/L | |  |
|  |  | mean |  | mean | Difference to placebo | P-value |
| Intra-erythrocytic creatine (µmol/L) |  |  |  |  |  |  |
| 3 weeks | 11 | 26 (-412, 464) |  | 623 (331, 916) | 598 (71, 1124) | 0.03 |
| 6 weeks | 11 | -100 (-569, 370) |  | 877 (564, 1189) | 976 (412, 1540) | 0.003 |
|  |  |  |  |  |  |  |
| Plasma creatine (µmol/L) |  |  |  |  |  |  |
| 3 weeks | 11 | 14 (-93, 121) |  | 140 (69, 211) | 101 (-131, 334) | 0.35 |
| 6 weeks | 11 | 48 (-146, 241) |  | 149 (21, 278) | 126 (-3, 254) | 0.054 |
