# Supplementary material for: Feasibility, safety and tolerability of intradialytic creatine supplementation in hemodialysis: A double-blind, randomized, placebo-controlled pilot study
Source: PLoS One. 2026 Aug 5;21(8):e0354883. doi: 10.1371/journal.pone.0354883 (PMC13440799; doi:10.1371/journal.pone.0354883)
Supplement: Supplementary file 12 [file pone.0354883.s012.docx]

| **S6 Table. Sensitivity analyses for primary outcomes with two lowest and two highest creatine groups pooled.** Results from sensitivity linear mixed model analyses: The effect of the study treatment for the two lowest and two highest dosage treatment groups of creatine supplementation pooled into two intervention groups on primary outcomes compared to placebo at 3 weeks and at 6 weeks of receiving the study intervention. | | | | | | | | | | | |
| --- | --- | --- | --- | --- | --- | --- | --- | --- | --- | --- | --- |
| Delta over time: | df |  | Placebo |  | 0.5 & 1.0 mmol/L |  |  |  | 1.5 & 2.0 mmol/L |  |  |
|  |  |  | mean |  | Mean | Difference to placebo | P-value |  | mean | Difference to placebo | P-value |
| Intra-erythrocytic creatine (µmol/L) |  |  |  |  |  |  |  |  |  |  |  |
| 3 weeks | 13 |  | 26 (-322, 373) |  | 233 (-50, 517) | 207 (-241, 656) | 0.34 |  | 792 (508, 1076) | 766 (317, 1214) | 0.003 |
| 6 weeks | 13 |  | -100 (-508, 309) |  | 442 (108, 776) | 541 (14, 1069) | 0.05 |  | 1022 (689, 1356) | 1122 (594, 1650) | <0.001 |
|  |  |  |  |  |  |  |  |  |  |  |  |
| Plasma creatine (µmol/L) |  |  |  |  |  |  |  |  |  |  |  |
| 3 weeks | 13 |  | 14 (-79, 107) |  | 42 (-34, 118) | 28 (-92, 148) | 0.62 |  | 171 (95, 247) | 156 (37, 277) | 0.014 |
| 6 weeks | 13 |  | 48 (-122, 218) |  | 51 (-88, 190) | 3 (-217, 223) | 0.98 |  | 189 (50, 328) | 141 (-78, 360) | 0.19 |
