# Supplementary material for: Feasibility, safety and tolerability of intradialytic creatine supplementation in hemodialysis: A double-blind, randomized, placebo-controlled pilot study
Source: PLoS One. 2026 Aug 5;21(8):e0354883. doi: 10.1371/journal.pone.0354883 (PMC13440799; doi:10.1371/journal.pone.0354883)
Supplement: Supplementary file 13 [file pone.0354883.s013.docx]

| **S7 Table. Secondary outcomes**. Results from linear mixed model analyses: The effect of the study treatment per treatment group on secondary outcomes compared to placebo at 3 weeks and at 6 weeks of receiving the study intervention. | | | | | | | | | | | | | | | | | | | | |
| --- | --- | --- | --- | --- | --- | --- | --- | --- | --- | --- | --- | --- | --- | --- | --- | --- | --- | --- | --- | --- |
| Delta over time: | df |  | Placebo |  | 0.5 mmol/L | |  |  | 1.0 mmol/L | |  |  | 1.5 mmol/L | |  |  | 2.0 mmol/L | |  | |
|  |  |  | mean |  | mean | efficacy* | p-value |  | mean | efficacy* | p-value |  | mean | efficacy* | p-value |  | mean | efficacy* | p-value | |
| **Functional measurements:** |  |  |  |  |  |  |  |  |  |  |  |  |  |  |  |  |  |  |  | |
| Hand grip strength (kg) | 11 |  | 0.5  (-4.0, 5.0) |  | 1.7  (-3.5, 6.8) | 1.7  (-5.6, 8.0) | 0.71 |  | 5.5  (0.3, 10.7) | 5.0  (-1.8, 11.8) | 0.14 |  | 0.7  (-4.5, 5.8) | 0.2  (-6.6, 7.0) | 0.96 |  | 1.0  (-4.2, 6.2) | 0.5  (-6.3, 7.3) | 0.88 | |
| Hand grip strength (%) | 11 |  | -0.9  (19.6, 17.8) |  | 6.2  (-15.4, 17.8) | 7.1  (-21.5, 35.7) | 0.6 |  | 29.0  (7.4, 50.6) | 29.9  (1.3, 58.5) | 0.04 |  | 2.9  (-19.7, 24.5) | 3.9  (-24.7, 32.4) | 0.77 |  | 3.7  (-17.9, 25.4) | 4.7  (-23.9, 33.2) | 0.73 | |
| FEV1 (L) | 8 |  | .12  (-0.29, 0.53) |  | -0.47  (-0.88, -0.6) | -0.59  (-1.17, 0.01) | 0.046 |  | -0.36  (-0.70, -0.03) | -0.48  (-1.00, 0.04) | 0.07 |  | 0.05  (-0.28, 0.38) | -0.07  (0.60, 0.46) | 0.07 |  | 0.047  (-0.29, 0.38) | -0.07  (-0.60, 0.45) | 0.76 | |
| FEV1 (%) | 8 |  | 2.5  (-12.4, 17.4) |  | -12.5  (-27.4, 2.4) | -14.9  (-36.0, 6.1) | 0.14 |  | -12.8  (-25.0, -0.7) | -15.3  (-34.6, 3.9) | 0.1 |  | 4.1  (-8.1, 16.3) | 1.6  (-17.6, 20.8) | 0.85 |  | 2.2  (-9.9, 14.4) | -0.3  (-19.5, 19.0) | 0.98 | |
| Sit to stand test (sec) | 10 |  | -1.4  (-25.0, 22.2) |  | -0.4  (-27.7, 26.9) | 1.0  (-35.1, 37.1) | 0.95 |  | 3.5  (-23.8, 20.7) | 4.8  (-31.3, 40.9) | 0.77 |  | 0.71  (-34.1, 32.7) | 0.69  (-40.3, 41.6) | 0.97 |  | 26.5  (-0.8, 53.8) | 27.9  (-8.2, 64.0) | 0.12 | |
| Sit to stand test (%) | 10 |  | -10.3  (-141.9, 120.2) |  | 6.4  (-144.3, 157.2) | 16.8  (182.6, 216.2) | 0.86 |  | 21.6  (-129.1, 172.4) | 32.0  (-167.4, 231.4) | 0.73 |  | -3.6  (188.2, 181.0) | 6.7  (-219.3, 232.8) | 0.95 |  | 140.8  (-9.9, 291.6) | 151.2  (-48.2, 350.6) | 0.12 | |
| 9 hole peg test (sec) | 4 |  | -0.2  (-5.5, 5.1) |  | 3.9  (-3.6, 11.3) | 4.0  (-5.1, 13.2) | 0.29 |  | 5.7  (-1.8, 13.1) | 5.9  (-3.3, 15.0) | 0.15 |  | 0.4  (-4.9, 5.7) | 0.6  (-6.9, 8.0) | 0.84 |  | -2.1  (-6.4, 2.2) | -2.0  (-8.8, 4.9) | 0.47 | |
| 9 hole peg test (%) | 4 |  | -0.6  (-21.2, 19.9) |  | 15.1  (-14.0, 44.1) | 15.7  (-19.9, 51.3) | 0.29 |  | 15.0  (-14.1, 44.0) | 15.6  (-20.0, 51.1) | 0.29 |  | 0.1  (-20.4, 20.6) | 0.72  (-28.3, 29.8) | 0.95 |  | -8.3  (-25.0, 8.5) | -7.7  (-34.2, 18.9) | 0.47 | |
| **Bio-impedance analyses:** |  |  |  |  |  |  |  |  |  |  |  |  |  |  |  |  |  |  |  | |
| Fat free mass (kg) | 8 |  | 3.7  (-4.4, 11.7) |  | 1.3  (-6.8, 9.3) | -2.4  (-13.8, 9.0) | 0.64 |  | 4.4  (-2.1, 11.0) | 0.8  (-9.6, 11.1) | 0.87 |  | 4.4  (-2.2, 10.9) | 0.72  (-9.6, 11.1) | 0.88 |  | -6.7  (-7.2, 5.9) | 4.3  (-14.7, 6.0) | 0.37 | |
| Fat free mass (%) | 8 |  | 4.0  (-13.4, 21.4) |  | 2.1  (-15.2, 19.5) | -1.9  (-26.4, 22.7) | 0.87 |  | 7.6  (-6.6, 21.8) | 3.6  (-18.8, 26.0) | 0.72 |  | 12.1  (-2.1, 26.3) | 8.1  (-14.3, 30.5) | 0.43 |  | -1.5  (-15.6, 12.7) | -5.4  (-27.9, 17.0) | 0.59 | |
| Skeletal muscle index | 8 |  | 0.05  (-1.45, 1.55) |  | 0.10  (-1.40, 1.60) | -0.05  (-2.17, 2.07) | 0.96 |  | 0.97  (-0.26, 2.19) | 0.92  (-1.02, 2.85) | 0.31 |  | 0.10  (-1.13, 1.33) | 0.05  (-1.89, 1.99) | 0.95 |  | -0.13  (-1.36, 1.09) | -0.18  (-2.12, 1.75) | 0.83 | |
| Skeletal muscle index (%) | 8 |  | -0.37  (-19.9, 19.2) |  | 1.0  (-18.6, 20.6) | 1.4 (-26.3, 29.1) | 0.91 |  | 12.8  (-3.2, 28.8) | 13.2  (-12.1, 38.4) | 0.26 |  | 1.6  (-14.4, 17.5) | 1.9  (-23.3, 27.2) | 0.87 |  | -2.0  (-17.9, 12.0) | -1.6  (-26.9, 23.7) | 0.89 | |
| Phase angle | 8 |  | 0.40  (-0.18, 0.98) |  | -0.10  (-0.68, 0.48) | -0.50  (-1.32, 0.32) | 0.2 |  | -0.07  (-0.54, 0.41) | -0.47  (-1.22, 0.89) | 0.19 |  | 0.13  (-0.34, 0.61) | -0.27  (-1.02, 0.49) | 0.44 |  | 0.37  (-0.11, 0.84) | -0.03  (-0.79, 0.72) | 0.92 | |
| Phase angle (%) | 8 |  | 5.7  (-7.1, 18.5) |  | -1.8  (14.6, 10.9) | -7.5.  (-25.6, 10.5) | 0.37 |  | -2.0  (-12.4, 8.5) | -7.7  (-23.1, 8.8) | 0.32 |  | 2.6  (-7.8, 13.0) | -3.1  (-19.6, 13.4) | 0.68 |  | 8.6  (-1.8, 19.0) | 2.9  (-13.6, 19.4) | 0.7 | |
| Fat mass (kg) | 8 |  | -0.8  (-6.7, 5.1) |  | -0.3  (-6.3, 5.6) | 0.5  (-8.0, 8.9) | 0.91 |  | 1.4  (-6.3, 3.4) | -0.6  (-8.3, 7.0) | 0.85 |  | 0.0  (-4.9, 4.8) | 0.8  (-6.9, 8.4) | 0.82 |  | 3.4  (-1.5, 8.3) | 4.2  (-3.5, 11.9) | 0.24 | |
| Fat mass (%) | 8 |  | 11.7  (-19.0, 42.4) |  | 0.9  (-31.7, 29.8) | -12.7  (-56.1, 30.8) | 0.52 |  | -11.1  (-36.2, 14.0) | -22.8  (-62.5, 16.8) | 0.22 |  | -0.8  (-25.8, 24.3) | -12.5  (-52.1, 27.2) | 0.49 |  | 16.9  (-8.2, 41.9) | 5.2  (-34.5, 44.8) | 0.77 | |
| Fat percentage (%) | 8 |  | 0.2  (-6.8, 7.1) |  | -0.7  (-7.7, 6.3) | -0.9  (-10.7, 9.0) | 0.85 |  | -2.6  (-8.3, 3.1) | -2.7  (-11.7, 6.3) | 0.51 |  | -0.2  (-5.9, 5.5) | -0.4  (-9.4, 8.6) | 0.92 |  | 3.6  (-2.1, 9.3) | 3.4  (-5.6, 12.4) | 0.41 | |
| Fat percentage (%) | 8 |  | 7.5  (-21.7, 36.6) |  | -1.9  (-31.0, 27.2) | -9.4  (-50.6, 31.8) | 0.61 |  | -13.8  (-37.6, 10.0) | -21.3  (-58.9, 16.3) | 0.23 |  | -2.8  (-26.6, 21.0) | -10.3  (-47.9, 27.4) | 0.55 |  | 12.2  (-11.6, 36.0) | 4.8  (-32.8, 42.4) | 0.78 | |
| Intra cellular water (L) | 8 |  | 1.8  (-1.3, 4.9) |  | 0.3  (-2.8, 3.4) | -1.5  (-5.9, 2.9) | 0.46 |  | 1.6  (-0.9, 4.2) | -1.7  (-4.2, 3.9) | 0.93 |  | 0.6  (-1.9, 3.2) | -1.2  (-5.2, 2.9) | 0.52 |  | -0.1  (-2.7, 2.4) | -1.9  (06.0, 2.1) | 0.3 | |
| Extra cellular water (L) | 8 |  | 0.6  (-7.4, 8.6) |  | 0.7  (-7.3, 8.7) | 0.1  (-11.2, 11.4) | 0.98 |  | -5.3  (-11.8, 1.2) | -5.9  (-16.2, 4.4) | 0.22 |  | 0.1  (-6.5, 6.6) | -0.5  (-10.8, 9.8) | 0.91 |  | -0.3  (-6.8, 6.2) | -0.9  (11.2, 9.4) | 0.84 | |
| Extra cellular water (%) | 8 |  | 2.5  (-44.6, 49.6) |  | 4.4  (-42.7, 51.5) | 1.9  (-64.7, 68.6) | 0.95 |  | -31.5  (-70.0, 6.9) | -34.0  (-94.8, 26.8) | 0.23 |  | 0.2  (-38.2, 38.7) | -2.2  (-63.1, 58.6) | 0.93 |  | -2.1  (-40.5, 36.4) | -4.5  (-65.4, 56.3) | 0.87 | |
| ECW ratio | 8 |  | -0.00  (-0.02, 0.01) |  | 0.01  (-0.00, 0.02) | 0.01  (-0.00, 0.03) | 0.1 |  | 0.01  (-0.00, 0.02) | 0.01  (-0.00, 0.03) | 0.1 |  | -0.01  (-0.02, 0.00) | -0.00  (-0.02, 0.01) | 0.9 |  | -0.00  (-0.01, 0.01) | 0.00  (-0.01, 0.02) | 0.9 | |
| ECW ratio (%) | 8 |  | -1.2  (-4.0, 1.6) |  | 2.2  (-0.5, 5.0) | 3.5  (-0.5, 7.4) | 0.08 |  | 1.8  (-0.5, 4.1) | 3.0  (-0.6, 6.6) | 0.09 |  | -1.3  (-3.6, 1.0) | -0.1  (-3.7, 3.5) | 0.96 |  | -0.9  (-3.2, 1.4) | 0.3  (-3.3, 3.9) | 0.84 | |
| *Efficacy: Difference compared to placebo | | | | | | | | | | | | | | | | | | | |  |
