# Supplementary material for: Feasibility, safety and tolerability of intradialytic creatine supplementation in hemodialysis: A double-blind, randomized, placebo-controlled pilot study
Source: PLoS One. 2026 Aug 5;21(8):e0354883. doi: 10.1371/journal.pone.0354883 (PMC13440799; doi:10.1371/journal.pone.0354883)
Supplement: Supplementary file 14 [file pone.0354883.s014.docx]

| **S8 Table. Exploratory outcomes.** Results from linear mixed model analyses: The effect of the study treatment per treatment group on exploratory outcomes compared to placebo at 3 weeks and at 6 weeks of receiving the study intervention. | | | | | | | | | | | | | | | | | | | | | |  |
| --- | --- | --- | --- | --- | --- | --- | --- | --- | --- | --- | --- | --- | --- | --- | --- | --- | --- | --- | --- | --- | --- | --- |
| Delta over time: | df |  | Placebo |  | 0.5 mmol/L | |  |  | 1.0 mmol/L | |  |  | 1.5 mmol/L | |  |  | 2.0 mmol/L | |  |  | Overall |  |
|  |  |  | mean |  | mean | efficacy* | p-value |  | mean | efficacy* | p-value |  | mean | efficacy* | p-value |  | mean | efficacy* | p-value |  | p-value |  |
| Creatine pool (g) |  |  |  |  |  |  |  |  |  |  |  |  |  |  |  |  |  |  |  |  |  |  |
| 3 weeks | 4 |  | -4.0  (-20.8, 12.9) |  | 10.1.  (-3.6, 23.9) | 14.1  (-7.6, 35.8) | 0.15 |  | 6.2  (-19.6, 23.1) | 10.2  (-13.6, 34.0) | 0.30 |  | missing |  |  |  | missing |  |  |  | 0.02 |  |
| 6 weeks | 5 |  | 6.0  (-4.9, 16.9) |  | 13.5  (4.6, 22.4) | 7.5  (-6.5, 21.6) | 0.23 |  | 9.1  (0.2, 18.0) | 3.1  (-10.9, 17.2) | 0.59 |  | missing |  |  |  | missing |  |  |  |  |  |
|  |  |  |  |  |  |  |  |  |  |  |  |  |  |  |  |  |  |  |  |  |  |  |
| Dialytic creatinine excretion (µmol/L) |  |  |  |  |  |  |  |  |  |  |  |  |  |  |  |  |  |  |  |  |  |  |
| 3 weeks | 11 |  | -0.4  (-1.9, 1.2) |  | 1.8  (0.0, 3.6) | 2.2  (-0.2, 4.6) | 0.06 |  | 3.0  (1.2, 4.8) | 3.3  (1.0, 5.7) | 0.01 |  | 0.9  (-0.9, 2.7) | 1.2  (-1.1, 3.6) | 0.27 |  | 5.2  (3.4, 7.0) | 5.6  (3.2, 7.9) | <0.001 |  | <0.001 |  |
| 6 weeks | 11 |  | 0.9  (-2.4, 4.1) |  | 2.2  (-1.5, 6.0) | 1.4  (-3.6, 6.4) | 0.56 |  | 3.9  (0.1, 7.6) | 3.0  (-2.0, 8.0) | 0.22 |  | -0.9  (-4.7, 2.9) | -1.8  (-6.8, 3.2) | 0.45 |  | 3.1  (-0.6, 6.9) | 2.3  (-2.7, 7.2) | 0.34 |  |  |  |
|  |  |  |  |  |  |  |  |  |  |  |  |  |  |  |  |  |  |  |  |  |  |  |
| Intra-erythrocytic guanidinoacetate (µmol/L) |  |  |  |  |  |  |  |  |  |  |  |  |  |  |  |  |  |  |  |  |  |  |
| 3 weeks | 11 |  | -0.17  (-0.67, 0.33) |  | 0.44  (-0.13, 1.02) | 0.62  (-0.14, 1.38) | 0.10 |  | -0.43  (-1.00, 0.15) | -0.25  (-1.02, 0.51) | 0.48 |  | -1.07  (-1.65, -0.49) | -0.90  (-1.66, -0.13) | 0.025 |  | -0.35  (-0.92, 0.23) | -0.17  (-0.94, 0.59) | 0.63 |  | 0.03 |  |
| 6 weeks | 11 |  | -0.40  (-0.94, 0.15) |  | -0.27  (-0.90, 0.36) | 0.13  (-0.71, 0.96 | 0.74 |  | -0.63  (-1.26, 0.00) | -0.23  (-1.06, 0.61) | 0.56 |  | -0.49  (-1.12, 0.14) | -0.09  (-0.93, 0.74) | 0.81 |  | -0.10  (-0.73, 0.53) | 0.30  (-0.53, 1.13) | 0.44 |  |  |  |
|  |  |  |  |  |  |  |  |  |  |  |  |  |  |  |  |  |  |  |  |  |  |  |
| Plasma guanidinoacetate (µmol/L) |  |  |  |  |  |  |  |  |  |  |  |  |  |  |  |  |  |  |  |  |  |  |
| 3 weeks | 11 |  | -0.19  (-0.56, 0.37) |  | -0.13  (-0.67, 0.40) | -0.04  (-0.75, 0.67) | 0.90 |  | -0.38  (-0.91, 0.16) | -0.28  (-1.00, 0.43) | 0.40 |  | -0.80  (-1.33,-0.26) | -0.70  (-1.42, 0.01) | 0.052 |  | -0.35  (-0.89, 0.19) | -0.26  (-0.97, 0.45) | 0.44 |  | 0.37 |  |
| 6 weeks | 11 |  | -0.20  (-0.68, 0.29) |  | -0.30  (-0.86, 0.26) | -0.11  (-0.85, 0.64) | 0.76 |  | -0.46  (-1.02, 0.11) | -0.26  (-1.01, 0.49) | 0.46 |  | -0.39  (-0.95, 0.17) | -0.19  (-0.94, 0.55) | 0.58 |  | -0.59  (-1.15, -0.02) | -0.39  (-1.14, 0.36) | 0.27 |  |  |  |
| *Efficacy: Difference compared to placebo | | | | | | | | | | | | | | | | | | | | | | |
