# Supplementary material for: Feasibility, safety and tolerability of intradialytic creatine supplementation in hemodialysis: A double-blind, randomized, placebo-controlled pilot study
Source: PLoS One. 2026 Aug 5;21(8):e0354883. doi: 10.1371/journal.pone.0354883 (PMC13440799; doi:10.1371/journal.pone.0354883)
Supplement: Supplementary file 15 [file pone.0354883.s015.docx]

| **S9 Table. Change in primary and exploratory outcomes during the 2-week washout period.** | | | | | | |
| --- | --- | --- | --- | --- | --- | --- |
|  | Delta visit 3 to visit 4, median (minimum - maximum) | | | | | |
| Group | Intra-erythrocytic creatine (µmol/L) | Plasma creatine (µmol/L) | Creatine pool (g) | Dialytic creatinine excretion (µmol/L) | Intra-erythrocytic guanidinoacetate (µmol/L) | Plasma guanidinoacetate (µmol/L) |
| Placebo | 63 (-334 - 327) | -69 (-260 - 2) | 7.2 (-4.6 - 19.6) | -0.8 (-2.6 - 2.6) | 0.28 (0.20 - 0.34) | -0.04 (-0.26 - 0.43) |
| 0.5 mmol/L | -79 (-172 - 169) | -23 (-93 - 44) | -6.0 (-20.9 - 3.1) | -0.9 (-1.5 - 0.5) | 0.55 (0.35 - 0.95) | 0.09 (-0.12 - 0.26) |
| 1.0 mmol/L | 25 (-533 - 225) | -64 (-150 - -14) | -10.6 (-15.7 - -5.8) | -2.5 (-4.8 - -1.0) | 0.28 (0.16 - 1.62) | 0.24 (-0.17 - 0.58) |
| 1.5 mmol/L | -864 - -350 | -346 - -111 | missing | -0.1 (-0.8 - 0.5) | 0.22 - 0.61 | 0.63 (0.35 - 0.90) |
| 2.0 mmol/L | -686 (-864 - -350) | -102 (-506 - -19) | -15.5 (missing) | -1.6 (-4.6 - 3.1) | 0.41 (0.05 - 0.52) | 0.22 - 0.31 |
