# Supplementary material for: Feasibility, safety and tolerability of intradialytic creatine supplementation in hemodialysis: A double-blind, randomized, placebo-controlled pilot study
Source: PLoS One. 2026 Aug 5;21(8):e0354883. doi: 10.1371/journal.pone.0354883 (PMC13440799; doi:10.1371/journal.pone.0354883)
Supplement: Supplementary file 16 [file pone.0354883.s016.docx]

| **S10 Table. Listing of all adverse events during the study period.** | | | | | | | |
| --- | --- | --- | --- | --- | --- | --- | --- |
| **Adverse event** | **Treatment group** | **Severity*** | **Frequency** | **When** | **Relatedness** | **Reasoning** | **Dropout** |
| Cardiac arrhythmia | Placebo | Moderate | 1x | After 4 weeks | Unlikely | Recurrence of AV nodal re-entry tachycardia (AVNRT). Verapamil dosages were being altered during the two months preceding study participation. Before the study participation, patient experienced AVNRT during dialysis twice in these two months. During one of the dialysis sessions in the study timeframe, the AVNRT recurred, which was captured on an electrocardiogram, and spontaneously converted back to sinus rhythm. Upon this event, the cardiologist started treatment with amiodaron.  This adverse event was considered unlikely to be related to study treatment, because participant received placebo and due to the course of events leading up to the study regarding previous arrythmia’s and changes in cardiologic medication. | No, but the study treatment duration was shortened, because the additional study procedures were burdensome for the patient in the context of this event. Visit 3 was completed ahead of time (at 4 weeks after baseline instead of 6), and visit 4 was completed 2 weeks after visit 3. Patient was included in the analyses. |
| Headache | Placebo | Mild | 1/week | Throughout the study period | Unlikely | This adverse event was considered unlikely to be related to study treatment, because the participant received placebo, and because there were no consistent complaints related to timing of study treatments. | Yes, due to personal reasons. |
| Headache | Placebo | Mild | 2-3x/week | Throughout the study period | Unlikely | This adverse event was considered unlikely to be related to study treatment, because participant received placebo, and because there were fluctuating periods of headaches during the study period, which were already present before commencement of the trial and persisted after finishing the trial. There were no consistent complaints related to timing of study treatments. | No |
| Dizziness | Placebo | Mild | 1x | At the 2nd treatment | Unlikely | This adverse event was considered unlikely to be related to study treatment, because although dizziness can be related to hemodynamic changes from hemodialysis itself, but unlikely due to addition of placebo fluid (water) to the dialysate. | Yes, due to personal reasons. |
| Leg stiffness | 1.0 mmol/L | Mild | 2-3x/week | Throughout the study period | Unlikely | Participant experienced leg stiffness several times during the study duration. This adverse event was considered unlikely to be related to study treatment, because there were no consistent complaints related to timing of study treatments, stiffness is not a known side effect of creatine, and there is no clear physiological mechanism, although this cannot entirely be ruled out. | No |
| Weight gain and fluid retention | 1.0 mmol/L | Moderate | 1x | After week 3. | Unlikely | This participant had severe aortic valve stenosis (AoS) and had been deemed unsuitable for transcatheter aortic valve replacement (TAVR) one month prior to study participation due to general frailty and comorbidities.  From the third week of receiving the study intervention, the participant gradually developed weight gain and pitting oedema. This was attributed to decreased diuresis secondary to forward failure in the context of severe aortic valve stenosis, while maintaining a high fluid intake, which the participant was used to. The treating team of the patient initiated furosemide in week 5, which was subsequently uptitrated over the following weeks. In addition, a fluid restriction was implemented. In week 7 ultrafiltration was used to further reduce fluid retention. With these treatments, the bodyweight of the patient gradually decreased.  This adverse event was considered unlikely to be related to the study treatment. Although mild fluid retention has been described as a potential side effect of creatine supplementation, this is attributed to a modest increase in intracellular water and would not typically result in clinically evident pitting oedema. In the case of this participant, there were signs of extracellular fluid retention in the setting of reduced diuresis due to AoS with forward failure and persistent high fluid intake. Therefore this adverse event was attributed to AoS and the patient was treated accordingly. | No, because the AE was attributed to AoS, and the patient was enthusiastic about the study participation. |
| Nausea | 1.0 mmol/L | Mild | 1x | At 2 weeks | Unlikely | This adverse event was considered unlikely to be related to study treatment, because the nausea occurred only once with a duration of 5 minutes during a treatment session, and not during the rest of the trial. | No |
| Hyperkalemia | 1.0 mmol/L | Moderate | 1x | At 2 weeks | Unlikely | Hyperkalemia of 7.2 mmol/L before starting a dialysis session after one week of study treatment. The hyperkalemia was corrected with dialysis.  This adverse event was considered unlikely to be related to study treatment, because hyperkalemia is a common adverse event in dialyzing patients with end stage kidney disease, and no other occurrences of severe hyperkalemia occurred in in this patient or in any of the other participants during the study. | No |
| Nausea and dysgeusia | 1.5 mmol/L | Mild | 3x | During first week, followed by drop-out | Likely | Complaints started within minutes after starting the dialysis session with creatine suppletion, which persisted approximately until 10 minutes after stopping creatine suppletion (while continuing dialysis treatment). Patient received treatment 3 times, all three times the complaints occurred, and intradialytic creatine suppletion was stopped after approximately one hour every time.  This adverse event was considered likely to be related to study treatment, because of the consistent temporal association with the study treatment, and gastrointestinal complaints are a known possible side effect of creatine. | Yes, due to the AE, after 1 week of study treatment. |
| Headache | 1.5 mmol/L | Mild | 1/week | Throughout the study period | Unlikely | This adverse event was considered unlikely to be related to study treatment, because there was no consistent temporal association with the study treatment. | No |
| Nausea | 2.0 mmol/L | Mild | 3x/week | Every dialysis session | Likely | Consistent complaints throughout the study period, starting during dialysis treatments.  This adverse event was considered likely to be related to study treatment, because of the consistent temporal association with the study treatment, and gastrointestinal complaints are a known possible side effect of creatine. | No, because the complaints were mild and the participant wished to continue with the study treatment. |
| *Severity was defined according to Common Terminology Criteria for Adverse Events. | | | | | | | |
