# Supplementary material for: Feasibility, safety and tolerability of intradialytic creatine supplementation in hemodialysis: A double-blind, randomized, placebo-controlled pilot study
Source: PLoS One. 2026 Aug 5;21(8):e0354883. doi: 10.1371/journal.pone.0354883 (PMC13440799; doi:10.1371/journal.pone.0354883)
Supplement: Supplementary file 18 [file pone.0354883.s018.docx]

**S2 Fig. Stability testing.** Results of the stability tests of the investigational product, showing creatine concentrations over time for the technical batches and the confirmation batch.
