# Supplementary material for: Feasibility, safety and tolerability of intradialytic creatine supplementation in hemodialysis: A double-blind, randomized, placebo-controlled pilot study
Source: PLoS One. 2026 Aug 5;21(8):e0354883. doi: 10.1371/journal.pone.0354883 (PMC13440799; doi:10.1371/journal.pone.0354883)
Supplement: Supplementary file 19 [file pone.0354883.s019.docx]

**S3 Fig. Creatine uptake.** A. Mean creatine uptake per treatment group per study visit. B. Mean creatine uptake per kg of fat free mass per group per study visit. C. Mean change in plasma creatine concentration per treatment group per study visit. Whiskers represent minimum and maximum values and p-values are based on an overall between-group comparison.
