# Supplementary material for: Feasibility, safety and tolerability of intradialytic creatine supplementation in hemodialysis: A double-blind, randomized, placebo-controlled pilot study
Source: PLoS One. 2026 Aug 5;21(8):e0354883. doi: 10.1371/journal.pone.0354883 (PMC13440799; doi:10.1371/journal.pone.0354883)
Supplement: Supplementary file 20 [file pone.0354883.s020.docx]

**S4 Fig. Exploratory outcomes.** The effect of different dosages of intradialytic creatine suppletion and placebo on exploratory study outcomes (A. Estimated total creatine pool. B. Dialytic creatinine excretion. C. Plasma guanidino acetate. D. Intra-erythrocytic guanidinoacetate) at three and six weeks of receiving the study intervention compared to baseline. The graphs show estimated marginal means derived from linear mixed model analyses with whiskers representing 95% confidence intervals.
