# Supplementary material for: Feasibility, safety and tolerability of intradialytic creatine supplementation in hemodialysis: A double-blind, randomized, placebo-controlled pilot study
Source: PLoS One. 2026 Aug 5;21(8):e0354883. doi: 10.1371/journal.pone.0354883 (PMC13440799; doi:10.1371/journal.pone.0354883)
Supplement: Supplementary file 21 [file pone.0354883.s021.docx]

**S5 Fig. Creatine excretion.** Urinary and dialytic creatinine excretion per study visit per individual participant. Placebo treated patients are marked with a “*”. Note that in participant 2, 6, 9, 10, 11, 14, 15 and 16 some urinary excretions could not be shown due to missing urine collections. Participant 5 and 8 had no residual diuresis. A. Creatinine excretion per dialytic interval (end of prior dialysis session to end of current dialysis session). B. Mean creatinine excretion per day (creatine excretion per dialytic interval devided by length of dialytic interval in days).
